# Functional Vascularization in Tumor Organoid and Tumor-on-Chip Models: Evidence Requirements for Attributing Therapy-Resistance Mechanisms

**DOI:** 10.3390/bioengineering13080947

**Published:** 2026-08-21

**Authors:** Yafang Li, Yiyi Yang, Lina Zhao, Zihao Bian, Zongjiu Zhang

**Affiliations:** Department of Biomedical Engineering, Tsinghua University, Beijing 100084, China; liyafangnku@163.com (Y.L.); yy-yang24@mails.tsinghua.edu.cn (Y.Y.); zhaoln23@mails.tsinghua.edu.cn (L.Z.); bianzh24@mails.tsinghua.edu.cn (Z.B.)

**Keywords:** vascularized tumor organoid, tumor-on-chip, therapy resistance, mechanism attribution, endothelial barrier, tumor microenvironment, microphysiological system, evidence appraisal

## Abstract

Vascularized tumor organoid and tumor-on-chip models are increasingly used to examine treatment response, yet a weak therapeutic effect does not by itself identify the failed transport or biological step. Reduced efficacy may result from inadequate delivery, altered endothelial transport or signaling, stromal protection, impaired immune access or function, tumor-intrinsic resistance, or several processes acting together. A structured literature search and narrative synthesis were combined with a focused qualitative appraisal of 25 eligible primary studies to determine what evidence is needed to distinguish these explanations. Twenty-one studies included a relevant comparator, a mechanism-directed perturbation, and a functional readout; four directly tested a principal competing explanation, and three included rescue or reversal. Most studies therefore supported a contributory mechanism under the tested conditions rather than an exclusive causal attribution, leaving transport, stromal, immune, and tumor-intrinsic routes incompletely separated. The synthesis defines five candidate failed-step classes and specifies the comparisons, local-exposure or step-resolved measurements, perturbations, orthogonal readouts, and alternative tests needed for each. Mechanistic attribution is most secure when the proposed bottleneck is measured directly, manipulated experimentally, and distinguished from credible alternatives. Clinical application will additionally require context-of-use-specific evidence of analytical reproducibility, reference treatments, prespecified acceptance criteria, and association with clinically relevant outcomes.

## 1. Introduction

Therapeutic response in a solid tumor reflects interacting cancer-cell and microenvironmental processes rather than tumor-cell sensitivity alone [1]. Therapeutic activity additionally depends on delivery, tissue transport, target engagement, and sustained biological activity [2]. Solid stress, extracellular-matrix organization, and stromal signaling can alter transport and cell survival [2,3,4]. Cell-based immunotherapies additionally require vascular adhesion, extravasation, stromal migration, target recognition, and maintained effector function [5]. Reduced efficacy in a multicellular model is therefore a composite endpoint that can be produced by several distinct failed steps [1,2,3,4,5].

Conventional model classes resolve different parts of this sequence. Two-dimensional cultures permit controlled exposure and targeted genetic or pharmacological perturbation, whereas tumor spheroids and patient-derived organoids retain three-dimensional organization and tumor-intrinsic heterogeneity [6,7,8,9]. Microphysiological systems add defined fluidic, material, and multicellular boundary conditions that can be configured for compartment-resolved transport and interaction studies [10,11,12,13,14,15].

Vascularized tumor organoid and tumor-on-chip models couple a three-dimensional tumor compartment to an endothelial interface or a microvascular network [16,17,18]. In the vascularized-organoid and organ-on-chip literature, vascular function is demonstrated through patent or perfusable lumens, measured barrier or transport behavior, or a measurable consequence for tissue support, therapeutic entry, or cell trafficking rather than through endothelial-marker expression alone [16,17,18]. Accordingly, eligibility for the focused appraisal required evidence of vascular function beyond endothelial-marker expression.

Recent reviews have established complementary engineering, biological, and pharmacological foundations for the field. Konopka and colleagues surveyed vascularized tumor-on-chip platforms for studying neovasculature and treatment response [19], whereas Kim and colleagues organized vascularized tumor–chip models around tumor–microenvironment–drug interactions [20]. Poljak and colleagues connected pathological tumor vasculature with perfusion engineering and therapeutic innovation [21], and Menzani and colleagues compared top-down, bottom-up, and hybrid strategies for vascularizing organoids on chips [22]. Wu and colleagues synthesized the cellular and molecular regulation of functional tumor-organoid vasculature [23]. Huang and colleagues examined how vascular transport, tissue exposure, endothelial state, and intrinsic sensitivity jointly shape therapeutic interpretation [24]. Together, these reviews establish the principal biological, engineering, and pharmacological variables that shape treatment response. What remains less clearly resolved is the evidence required to attribute a weak treatment effect to one mechanism rather than another at the level of an individual experiment, and the additional test needed when credible alternatives remain. Table 1 compares the organizing emphases of these reviews with the focus of the present analysis.

Mechanism attribution is difficult because the same terminal endpoint can arise from failures at different points along the vascular-to-tumor sequence. Observation of a weak response is therefore insufficient. A mechanism-specific interpretation requires a comparison that differs in the proposed bottleneck, measurement of the relevant exposure or biological step, experimental perturbation of the proposed cause, and assessment of credible alternatives. This approach is consistent with established principles of causal and mechanistic inference, including intervention on the proposed cause, temporally and spatially resolved measurement, consideration of alternative explanations, and convergence across methods [25,26,27]. The appraisal therefore records experimentally testable features rather than assigning a universal study-quality score [26].

**Figure 1:**
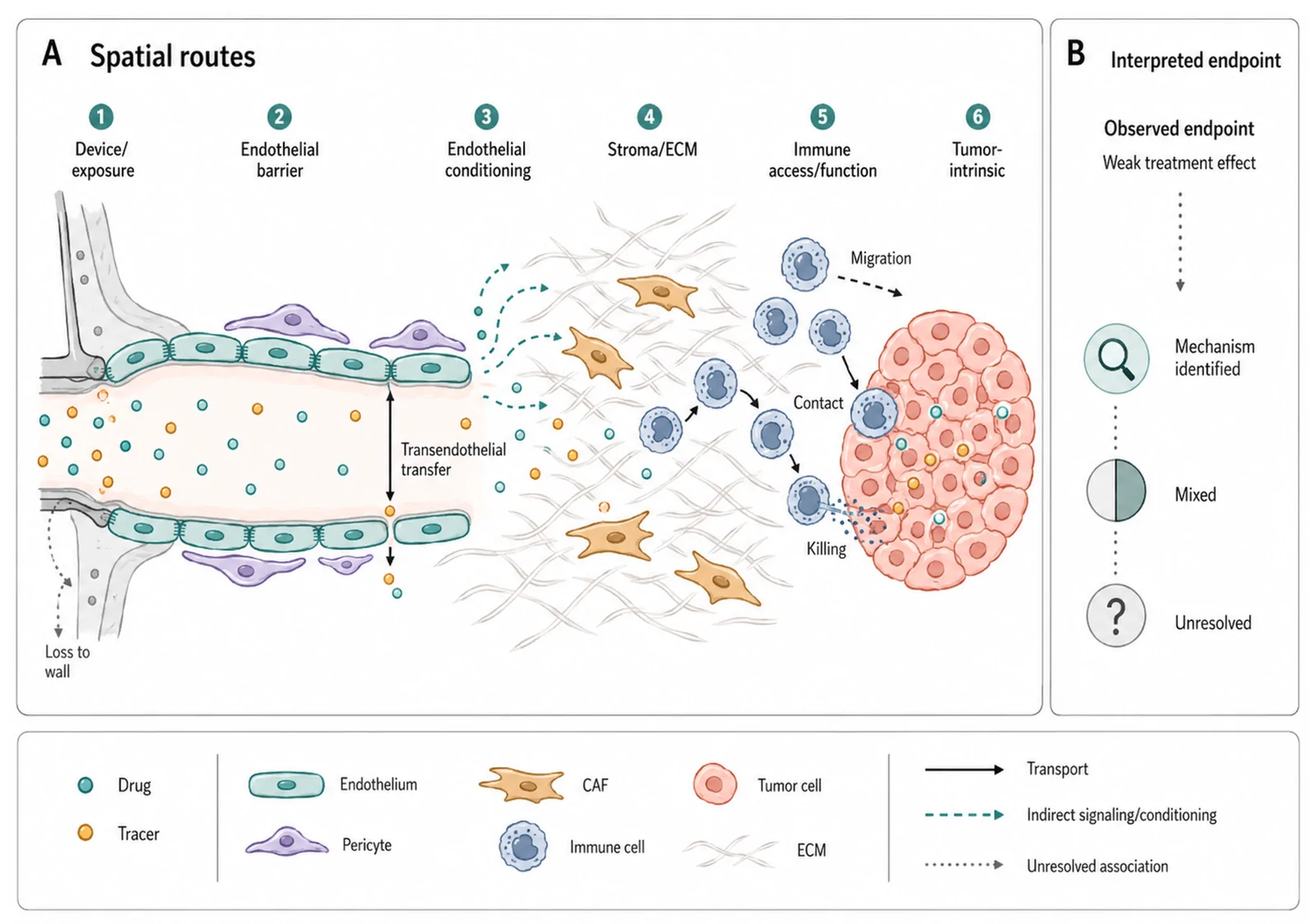
Spatial decomposition of a weak treatment effect into competing failed steps. (**A**) The same terminal endpoint may arise from device or vascular exposure loss, an endothelial barrier, endothelial conditioning, stromal or extracellular-matrix protection, immune-access or post-contact failure, tumor-intrinsic resistance, or a coupled combination. Solid arrows indicate transport or directed movement, teal dashed arrows indicate indirect signaling or conditioning, and gray dotted paths indicate unresolved backtracking associations. (**B**) A weak endpoint supports a mechanism-specific conclusion only when the failed step is identified; otherwise, the result remains mixed or unresolved. The illustration is conceptual and contains no experimental measurements or quantitative encoding. CAF, cancer-associated fibroblast; ECM, extracellular matrix.

Accordingly, the mechanism-specific conclusion, rather than the platform type, serves as the unit of analysis. Weak treatment response is decomposed into five candidate failed-step classes: device or vascular exposure failure; endothelial barrier or conditioning; perivascular, fibroblast, or extracellular-matrix protection; immune-access or post-contact dysfunction; and tumor-intrinsic resistance after exposure matching. For each class, the analysis specifies the comparator, measurement, perturbation, orthogonal readout, alternative test, and, where feasible, rescue or reversal needed to support the strongest defensible conclusion. Applying these requirements to 25 eligible primary studies identifies where the evidence supports a contributory mechanism and where transport, stromal, immune, and tumor-intrinsic explanations remain coupled. The synthesis provides a practical basis for experimental design, manuscript evaluation, and fit-for-purpose platform validation while preserving the distinction between mechanistic interpretation and clinical readiness. Figure 1 summarizes the central analytical problem.

## 2. Review Scope, Literature Search, and Qualitative Evidence Synthesis

### 2.1. Review Design and Literature Search

A structured literature search and narrative synthesis were conducted. Narrative synthesis was appropriate because device architecture, cell source, matrix formulation, therapeutic modality, exposure route, and endpoint definitions were too heterogeneous for a meaningful pooled effect estimate. The analysis compared mechanistic interpretations and translational requirements rather than estimating intervention effects or the prevalence of platform types. To support transparency, the objective, information sources, selection criteria, and basis for scientific reasoning are reported in accordance with established quality domains for narrative reviews [28].

Searches were conducted in PubMed, Web of Science, Scopus, and Google Scholar; the final search update was completed on 31 July 2026, with database-specific search dates reported in Supplementary Table S1A. The searches were supplemented by publisher records and backward and forward citation tracking. Search concepts combined vascularized or perfused tumor models with organoid-on-chip, tumor-on-chip, microphysiological-system, drug-delivery, therapy-response, resistance, immunotherapy, T-cell-trafficking, CAR-T, matched-comparator, local-exposure, mechanism-directed-perturbation, rescue, target-engagement, and orthogonal-readout terms. Database-specific strategies are provided in Supplementary Table S1A.

### 2.2. Eligibility, Study Selection, and Data Extraction

Eligible primary reports had a verifiable bibliographic identity and either met the Core evidence criteria or directly resolved an adjacent mechanistic or platform step. Core evidence required a three-dimensional tumor construct, an integrated endothelial or vascular compartment, and a mechanism-relevant treatment, transport, vascular-function, or immune-function endpoint. Boundary evidence lacked at least one Core element but directly informed an adjacent step, such as stromal protection without vascular entry or post-contact immune dysfunction without an endothelial access phase. Reviews, perspectives, duplicate reports, unverifiable records, and studies remote from the specified mechanisms were excluded. Application of these criteria yielded 25 studies: 18 Core and seven Boundary. These studies constitute the focused appraisal subset of the broader literature base used for the narrative synthesis. Detailed selection rules are provided in Supplementary Table S1B, and the scope-classification logic is summarized in Supplementary Figure S1.

Eligibility was assessed against the prespecified criteria, and study characteristics were extracted using a structured data-extraction scheme. Source records and study-level extraction data were retained to permit verification. Extracted fields included model composition, fabrication and fluidic configuration, therapeutic route, comparator, mechanism-directed perturbation, local exposure or target engagement, orthogonal readouts, rescue or reversal, competing explanations, replication, quality-control criteria, and clinical-validation features. Unreported items were retained as “not reported” or “unclear”. Bibliographic identity, publication status, DOI, and linked corrections were checked against publisher or indexed records. The complete extraction matrix is provided in Supplementary Table S2A,B.

### 2.3. Prespecified Evidence Features, Mechanism-Attribution Categories, and Translation Criteria

The appraisal did not assign an ordinal study-quality grade. For descriptive purposes, the extracted evidence was summarized using two independent categories developed for this synthesis. The mechanism-attribution category comprised four levels. M0 denoted structural feasibility only. M1 denoted an association consistent with the proposed mechanism but not specific to it. M2 required a mechanism-directed perturbation that changed the relevant outcome. M3 required discrimination of the proposed mechanism from the principal tested alternative. Main M3 required direct testing of the principal competing explanation; strict M3 additionally required rescue or reversal where technically feasible. These categories describe the supported inference and do not constitute risk-of-bias grades or overall study-quality scores.

The underlying experimental features were informed by established approaches to causal and mechanistic reasoning, which emphasize intervention on the proposed cause, step-specific measurement, consideration of alternatives, and convergence across observations with different limitations [25,26,27]. The recorded features were (1) a comparator that differed in the proposed bottleneck; (2) measurement of local exposure, target engagement, or the relevant biological step; and (3) a mechanism-directed engineering, cellular, genetic, or pharmacological perturbation. Additional features were (4) a step-resolved or orthogonal functional readout; (5) direct testing of the principal competing explanation; and (6) rescue or reversal where feasible. Each feature was recorded as present, absent, or unclear; no composite numerical quality score was calculated.

**Figure 2:**
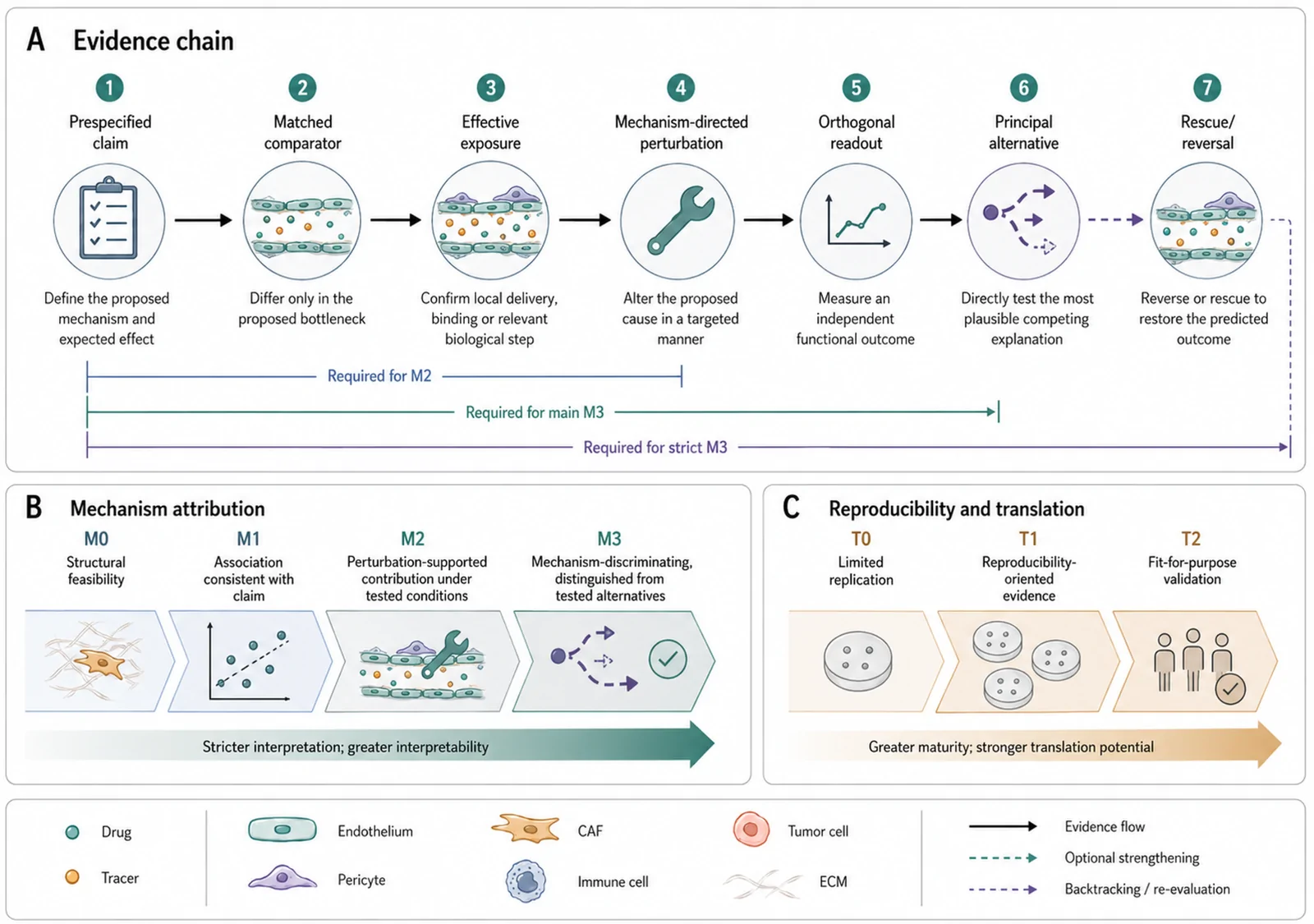
Evidence sequence and independent descriptive categories. (**A**) A mechanism-specific inference proceeds from a prespecified claim through a bottleneck-matched comparator, confirmation of effective exposure or the relevant biological step, a mechanism-directed perturbation, an orthogonal functional readout, direct testing of the principal alternative, and, where feasible, rescue or reversal. (**B**) M0–M3 describe the strongest mechanism-attribution claim supported by the experiment, from structural feasibility to discrimination from tested alternatives. Main M3 requires direct testing of the principal alternative; strict M3 additionally requires rescue or reversal where feasible. (**C**) T0–T2 describe a separate progression from limited replication to reproducibility-oriented evidence and fit-for-purpose validation. M and T are assessed independently and are not overall study-quality rankings.

The independent reproducibility and translation category comprised three levels. T0 denoted limited replication or single-site evidence. T1 required a reproducibility-oriented feature, such as independent repeats, multi-donor or multi-batch testing, prespecified quality control, or an external benchmark. T2 denoted fit-for-purpose validation for a defined context of use through cross-site, prospective, threshold-based, or comparable validation. M and T were assessed separately: a mechanistically discriminating experiment may remain immature for translation, whereas a reproducible platform may not distinguish a specific mechanism. Definitions and study assignments for the underlying evidence components are provided in Supplementary Table S3A,B. Figure 2 summarizes the evidence sequence and the independent M and T categories.

Generative-AI tools were used as author-assistance tools during manuscript preparation and revision for initial structuring of ideas, organization of draft material, preliminary conceptual-figure drafting, and language polishing. All literature selections, study classifications, evidence coding, reference verification, scientific interpretations, and final figure content were reviewed and finalized by the authors. Product details are provided in the Acknowledgments.

## 3. Fabrication and Operational Engineering as Determinants of Mechanistic Interpretability

Fabrication and operating conditions establish the route by which a therapeutic agent, tracer, or immune cell reaches the modeled tumor and thus determine which biological claims the experiment can resolve. Material selection can alter compound recovery and osmolality [29,30,31,32,33]; fabrication route and vascular geometry can alter channel dimensions, interface area, network connectivity, and transport behavior [34,35,36,37,38]; and fluidic initialization can introduce bubble-related shear, leakage, or loss of patency [39,40]. These engineering variables may change the measured treatment response before biological resistance can be inferred. Explicit specification, measurement, and qualification are therefore integral to the experimental design rather than ancillary technical details.

**Figure 3:**
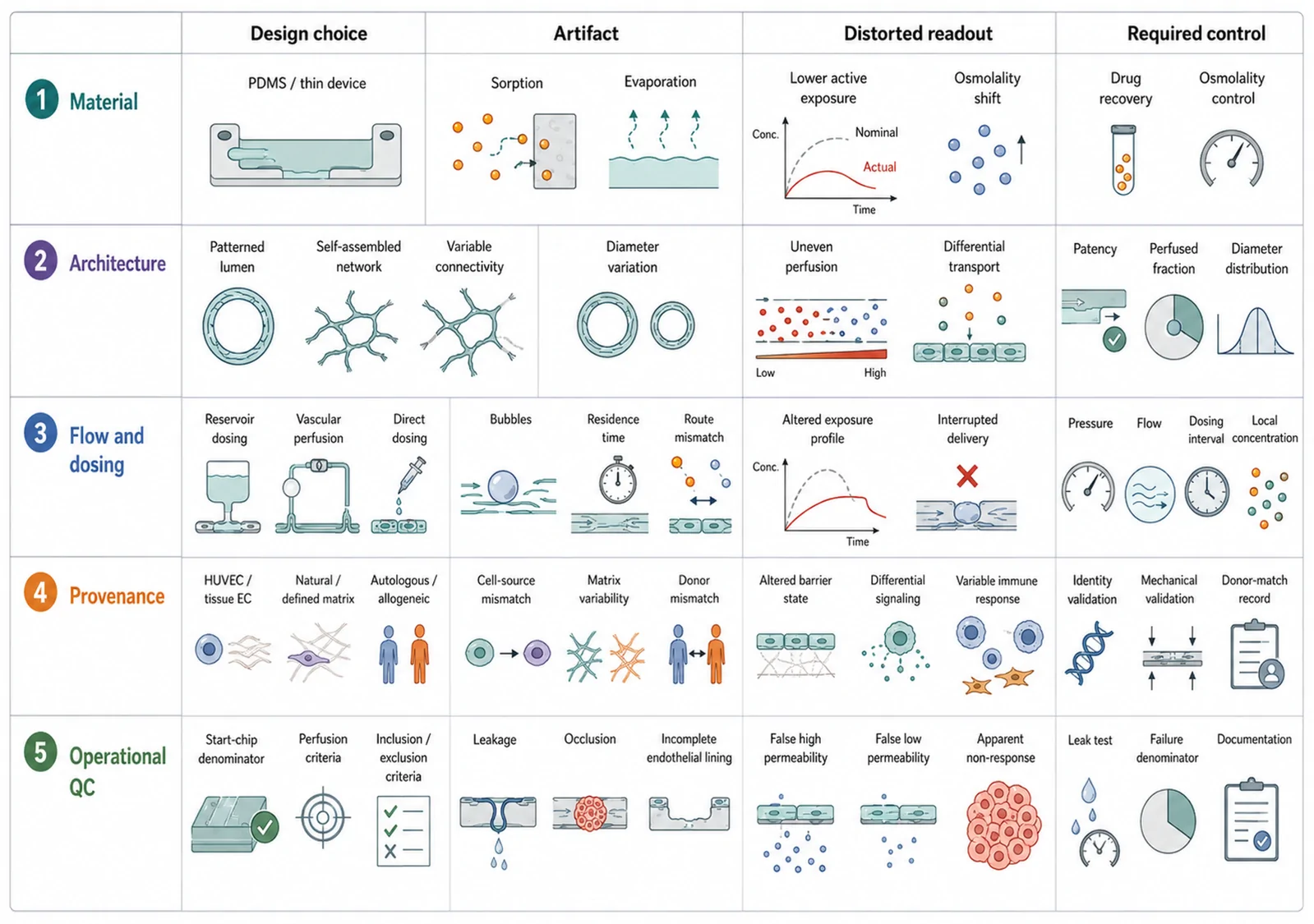
Engineering boundary conditions and controls for mechanistic interpretation. The five rows cover material, architecture, flow and dosing, biological provenance, and operational quality control. Across the four columns, each design choice is linked to a potential artifact, the readout that artifact can distort, and a corresponding control or reporting requirement. The diagram is conceptual and does not encode frequencies, effect sizes, or study rankings. EC, endothelial cell; HUVEC, human umbilical vein endothelial cell; PDMS, polydimethylsiloxane; QC, quality control.

Figure 3 summarizes the principal engineering choices, the artifacts they can introduce, the readouts they can distort, and the minimum controls required for interpretation.

### 3.1. Fabrication Choices Define the Interpretable Biological Question

The appropriate fabrication strategy depends on the proposed mechanistic bottleneck rather than on platform familiarity alone. An exposure-limited claim requires a circuit in which material recovery, dosing route, residence time, and concentration within the relevant compartment can be measured or bounded. An endothelial-barrier claim requires a stable interface with known geometry and a transport measurement that can be distinguished from network patency or route failure. A stromal or matrix claim requires composition, geometry, and mechanical state to be varied independently of perfusion, whereas an immune-access claim requires a defined vascular entry route and, where feasible, a direct-contact condition that bypasses that route [29,30,31,32,33,37,38,41,42,43,44,45]. A single device configuration cannot resolve every resistance hypothesis: compartment arrangement and measurement access determine which competing explanations can be separated experimentally.

Fabrication can also generate technical effects that mimic the biological mechanism under investigation. Material sorption or evaporation can lower active exposure; dimensional variation can alter hydraulic resistance and shear; and bonding defects, gel displacement, incomplete endothelial lining, occlusion, or bubbles can redirect or interrupt delivery [29,30,31,32,33,34,35,36,37,38,39,40]. A terminal viability or response difference may remain technically measurable under these conditions, but its mechanism cannot be assigned securely. Each design choice therefore needs a prospective link to the artifact it may introduce, the readout that artifact could distort, and a predefined control or run-acceptance criterion. Technical route failure must be excluded before a weak response is interpreted as endothelial, stromal, immune, or tumor-intrinsic resistance.

Technical and biological qualification address related but distinct questions. Technical qualification establishes that the device and fluidic circuit operate within stated tolerances; biological qualification establishes that the proposed pathway is measured and can be perturbed under those conditions. Automation, plate compatibility, and batch control can improve reproducibility, but they cannot replace a mechanism-matched comparator or a measurement of effective exposure. Added biological complexity likewise cannot compensate for an unqualified delivery route. For translational use, fabrication specifications need to match the context of use, reference treatments, acceptance thresholds, turnaround time, and unit of inference [40,46,47,48,49]. This alignment defines the engineering boundary within which a biological conclusion remains defensible.

### 3.2. Material Selection, Sorption, and Evaporation

Polydimethylsiloxane (PDMS) remains widely used because it supports rapid prototyping, optical access, gas exchange, and multilayer fluidics [31,32]. Hydrophobic small molecules can nevertheless partition into PDMS, reducing recovered and locally available concentrations independently of endothelial or matrix transport [29,30,31,32]. Water loss through thin PDMS components can increase medium osmolality during small-volume culture and thereby alter cell viability or phenotype [33]. These processes are distinct: a material-recovery experiment addresses sorption [29,30], whereas humidity, reservoir-volume, and osmolality controls address evaporation [33]. A reduced treatment effect in a PDMS device cannot be attributed securely to a vascular barrier without a material-only recovery control and a measurement or validated proxy of local exposure [29,30,31,32,33].

Thermoplastics, glass, and hybrid devices can reduce selected sorption or evaporation liabilities, but they introduce their own constraints in bonding, surface modification, optical access, gas transfer, geometric tolerance, and cell attachment [31,32]. Material selection therefore requires justification in relation to the therapeutic cargo, exposure duration, analytical readout, and intended context of use rather than by reference to a universally superior platform.

### 3.3. Fabrication Route, Geometry, Bonding, and Interface Integrity

Soft lithography, micromilling, molding, laser cutting, three-dimensional printing, bioprinting, and sacrificial templating produce different channel dimensions, surface roughness, alignment accuracy, optical access, and opportunities for closed fluidic connection [34,35,36]. Patterned lumens facilitate geometry-matched barrier comparisons and direct pressure or permeability measurements, whereas self-assembled networks reproduce capillary-scale branching and remodeling but introduce variability in connectivity, lumen diameter, and flow distribution [37,38]. The fabrication route consequently constrains both the modeled biology and the inference that can be drawn from it.

Bonding and hydrogel confinement are equally relevant to interpretation. Changes in channel height or width alter hydraulic resistance and wall shear [37,38]; incomplete bonding, leakage, delamination, gel displacement, or interface contraction can alter the delivered route and effective exposure [37,38,39,40]. When these variables can affect the proposed mechanism, reporting needs to include critical dimensions, tolerances, bonding or sealing method, production batch, sterilization procedure, and leak or interface acceptance tests [40]. In the absence of these records, apparent biological variation may instead reflect manufacturing or assembly variation.

### 3.4. Fluidic Initialization, Operational Stability, and Failure Reporting

Priming and perfusion must be established before a run can be interpreted biologically [37,38,39,40]. A gas bubble can produce a local shear perturbation and compromise cell viability or function in a perfused microchannel [39]. Incomplete priming, channel occlusion, interrupted flow, unstable pressure, leakage, and incomplete endothelial coverage can produce heterogeneous or absent exposure [37,38,39,40]. These events are prespecified technical quality-control outcomes rather than post hoc evidence of treatment resistance.

The minimum operational record includes the number of initiated and accepted chips, prespecified exclusion criteria, failure categories, flow or pressure settings, connected perfused fraction or patency, barrier acceptance criteria where applicable, and assay failure rate [37,38,40]. Reporting only representative successful chips obscures whether the observed phenotype is reproducible at the level of the device run. A failed exposure route renders a mechanism-specific biological conclusion uninterpretable even when the terminal viability measurement is technically available.

### 3.5. Manufacturability, Batch Control, and Scale-Up

Mechanistic interpretability and manufacturability are related but separate performance dimensions. Plate-compatible formats, closed fluidics, standardized connectors, automated loading, and predefined acceptance criteria can reduce operator-dependent variation and support larger experimental series [40]. They do not, by themselves, establish biological validity or clinical utility. Conversely, a mechanistically informative prototype may remain unsuitable for broader use if its geometry, surface treatment, cell loading, or perfusion cannot be reproduced across production batches.

For each stated context of use, reporting needs to distinguish technical replicates from independent device batches and identify the unit used for inference. Batch identifiers, dimensional verification, material and coating lots, sterilization, storage, assembly yield, and acceptance thresholds are particularly relevant when a platform is proposed for cross-site testing or standardized deployment [40]. This fabrication-level record establishes whether subsequent differences can reasonably be assigned to biology rather than to production or operation.

## 4. Biological and Experimental Boundary Conditions That Shape Mechanistic Inference

After fabrication and operational integrity have been established, biological composition and exposure geometry determine which mechanistic questions the model can resolve. Interpretation depends on aligning vascular architecture, endothelial provenance, dosing route, matrix properties, stromal and immune-cell state, and functional readouts with the proposed failed step rather than treating these features as generic markers of model complexity.

### 4.1. Vascular Architecture and Endothelial Provenance

Vascularized models range from planar endothelial barriers and patterned lumens to angiogenic sprouts and self-assembled microvascular networks [22,37,38,40]. Patterned vessels provide reproducible geometry, defined surface area, and direct access to pressure or permeability measurements [37,38]. Self-assembled networks provide branched microvessels and stromal interactions but can vary in connectivity, lumen diameter, and flow distribution [22,35,36,37,38,40]. The critical question is whether the architecture reproduces and measures the bottleneck specified by the claim, rather than whether the model is simply described as vascularized.

Endothelial provenance also limits the supported inference. Human umbilical vein endothelial cells (HUVECs) are accessible and can form perfusable networks, but they do not reproduce every tissue-specific microvascular phenotype [37,38,41]. In a direct microfluidic comparison, brain microvascular endothelial-cell networks exhibited lower dextran permeability than HUVEC networks, demonstrating that endothelial source can alter engineered barrier function [41]. Endothelial sources can also differ in microvascular formation and barrier permeability [41]. Claims about organ-specific inflammatory, transport, or angiocrine behavior therefore require the corresponding phenotype to be measured rather than inferred from the cell-source label alone [37,38,41]. Mural-cell coverage and basement-membrane deposition likewise require reporting as functional variables rather than universal indicators of model quality [37,38,41].

### 4.2. Flow, Dosing Route, and Exposure Geometry

The administered dose does not necessarily represent the concentration-time profile within the tumor compartment [29,30,31,32,37,42,43]. Direct addition to an open reservoir, premixing within a hydrogel, injection into a vascular channel, and continuous perfusion generate different exposure histories [37,42,43]. Flow rate and wall shear stress can alter endothelial permeability and tumor-endothelial angiogenic signaling in a microfluidic tumor-vascular model [42]. Pressure gradient, residence time, sampling schedule, and washout further influence delivery [37,42,43]. Therapeutic route, timing, flow, and recovery form part of the biological exposure definition and require reporting rather than treatment solely as device settings [37,42,43].

For a small molecule, the relevant sequence can include device loss, vascular concentration, transendothelial transfer, matrix diffusion, nonspecific binding, and intracellular target engagement [29,30,31,32,37,43]. For an antibody, nanoparticle, or cell therapy, size, deformability, receptor-mediated transport, margination, adhesion, and viability become more prominent [5,37,43]. A tracer supports a therapeutic-delivery inference only when its size, charge, binding, and transport behavior are relevant to the cargo [29,30,31,32,37,43]. Fluorescent dextran can characterize barrier permeability, and fluorescein can demonstrate wash-in or wash-out, but neither alone establishes the distribution of a hydrophobic drug, antibody, nanoparticle, or cellular product [29,30,31,32,37,43].

### 4.3. Matrix, Mural-Cell, Stromal, and Immune Provenance

Natural matrices support survival and morphogenesis but can introduce poorly defined biochemical composition and limited independent control of mechanics, degradability, ligand density, and transport [50]. Fully synthetic matrices permit more explicit control of these parameters and have supported human organoid culture across donors and tissue types, although they do not automatically reproduce every function obtained in Matrigel or tissue-derived matrices [50]. Neither material class is universally preferable. A matrix-limited-delivery claim requires a transport or penetration measurement and a record of the relevant matrix property; a mechanotransduction claim requires mechanical characterization and a pathway-level response.

Pericytes, fibroblasts, and immune cells are functional variables rather than interchangeable additions. Cancer-associated fibroblasts (CAFs) include inflammatory, myofibroblastic, antigen-presenting, and other states with distinct secretomes, matrix effects, and vascular interactions [51,52,53]. Pericytes can modify tumor-cell response and contribute to a protective niche [54]. In vascular models, barrier stabilization and paracrine protection are separate hypotheses. Engineered immune-cell products are evaluated against defined antigen contexts, and their source, engineering protocol, viability, donor matching, and antigen context delimit the supported conclusion [55,56].

### 4.4. Functional and Orthogonal Readouts

A structural vascular image does not establish transport function [16,17,18,37,38]. Vascular readouts need to match the proposed mechanism and may include connected perfused fraction, permeability coefficient, lumen stability, flow distribution, pressure, and wall-shear conditions [37,38,43]. Drug-delivery studies require a local penetration, concentration, recovery, or target-engagement measurement in addition to the administered dose [29,30,31,32,37,43]. Immune studies require step-resolved measures of adhesion, arrest, extravasation, stromal migration, tumor contact, and cytotoxicity [44,45,55,56]. Terminal viability remains an important outcome, but it cannot identify the failed step in a sequential process [43,44,45,55,56].

**Figure 4:**
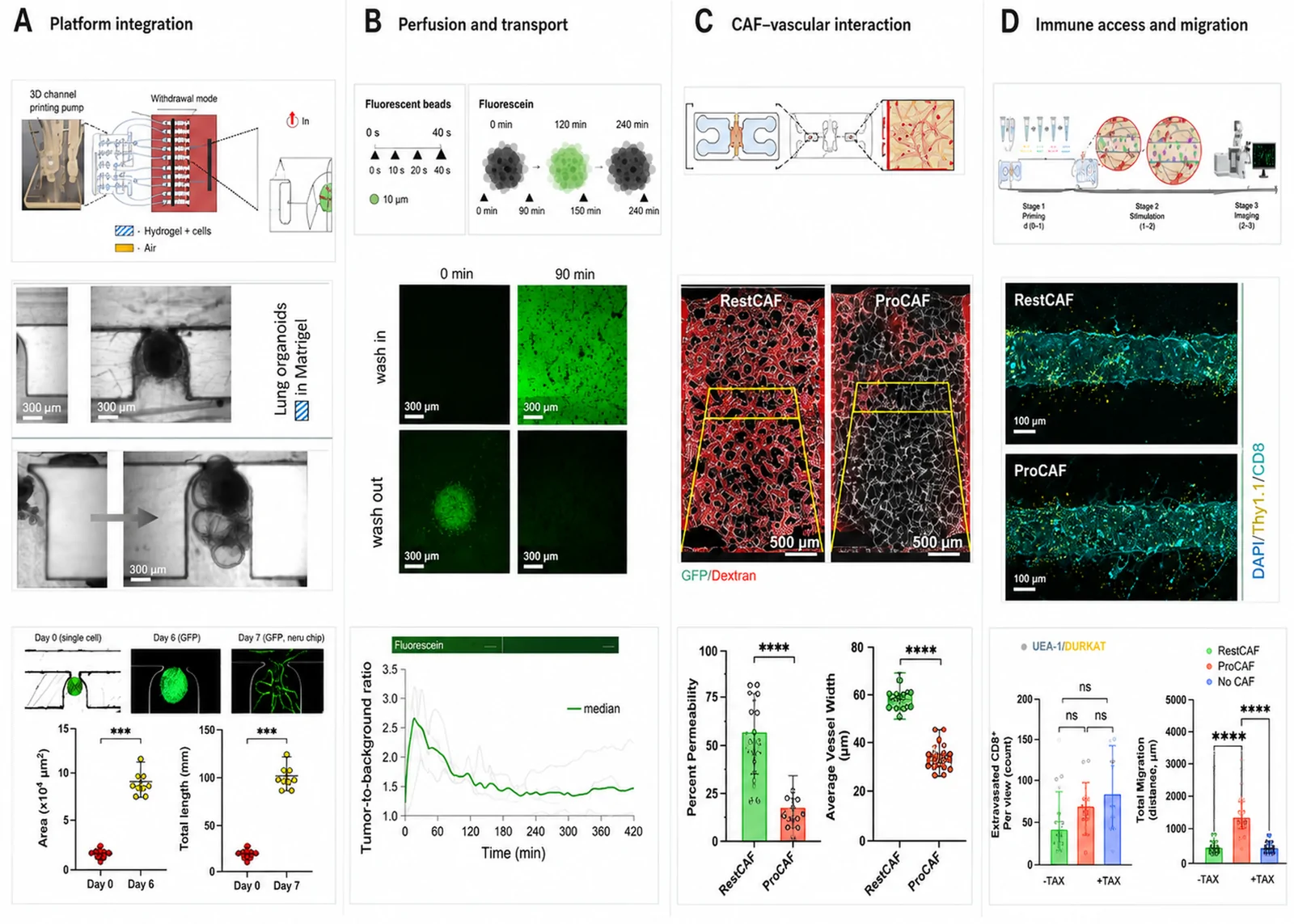
Representative experimental implementations and functional readouts arranged as four study stories. (**A**) Platform architecture, organoid trapping, and flow-associated endothelial-network development adapted from Quintard et al. (2024) [40], from original Figures 1a–d,h and 2a,b. These panels support device integration and vascularized-tissue implementation but not a tumor-specific therapy mechanism. (**B**) Fluorescent-bead perfusion, fluorescein wash-in and wash-out, and retention kinetics adapted from Skubal et al. (2025) [43], from original Figure 3A–G. These panels support vessel patency, convective transport, and solute-retention measurements; beads and fluorescein are proxies and do not establish therapeutic-drug exposure. (**C**) RestCAF- and ProCAF-associated microvascular networks, perfusability, and vessel-width measurements adapted from Blackledge et al. (2026) [59], from original Figure 2a–e,g,h. These panels support CAF-state-associated differences in vascular architecture and function but not a universal stromal resistance mechanism. (**D**) Jurkat T-cell extravasation and migration adapted from Blackledge et al. (2026) [59], from original Figure 6a,c(i–iv). These panels distinguish access-related readouts from post-contact effector function and do not demonstrate tumor killing. The selected source articles are distributed under the Creative Commons Attribution 4.0 International License. Panels were cropped, proportionally resized, arranged, and relabeled; original scale bars, axes, units, data points, error bars, and statistical annotations were retained where present. In panel A, *** denotes p<0.001 as reported by Quintard et al.; in panels (**C**,**D**), **** denotes p<0.0001 as reported by Blackledge et al.; ns denotes not significant. CAF, cancer-associated fibroblast; RestCAF and ProCAF denote the CAF states named in the source study.

Longitudinal imaging, sensors, secretome profiling, and molecular assays provide orthogonal evidence only when they are linked to the proposed bottleneck [57,58]. Image-analysis methods can improve reproducibility, but morphological predictions require biological labels and validation against functional measurements [57,58]. Tronolone and colleagues used supervised machine learning to relate vascular-network morphology to oxygen-transport function, illustrating that morphological predictions require validation against biological performance rather than interpretation in isolation [57,58]. Representative device, tissue, transport, stromal, and immune-access readouts are shown in Figure 4. Supplementary Figure S2 provides additional mechanism-focused examples in which a published image is directly paired with its functional readout. Table 2 summarizes the associated risks of misinterpretation, minimum controls, and strongest defensible conclusions.

## 5. Evidence Requirements for Attributing Therapy-Resistance Mechanisms

**Figure 5:**
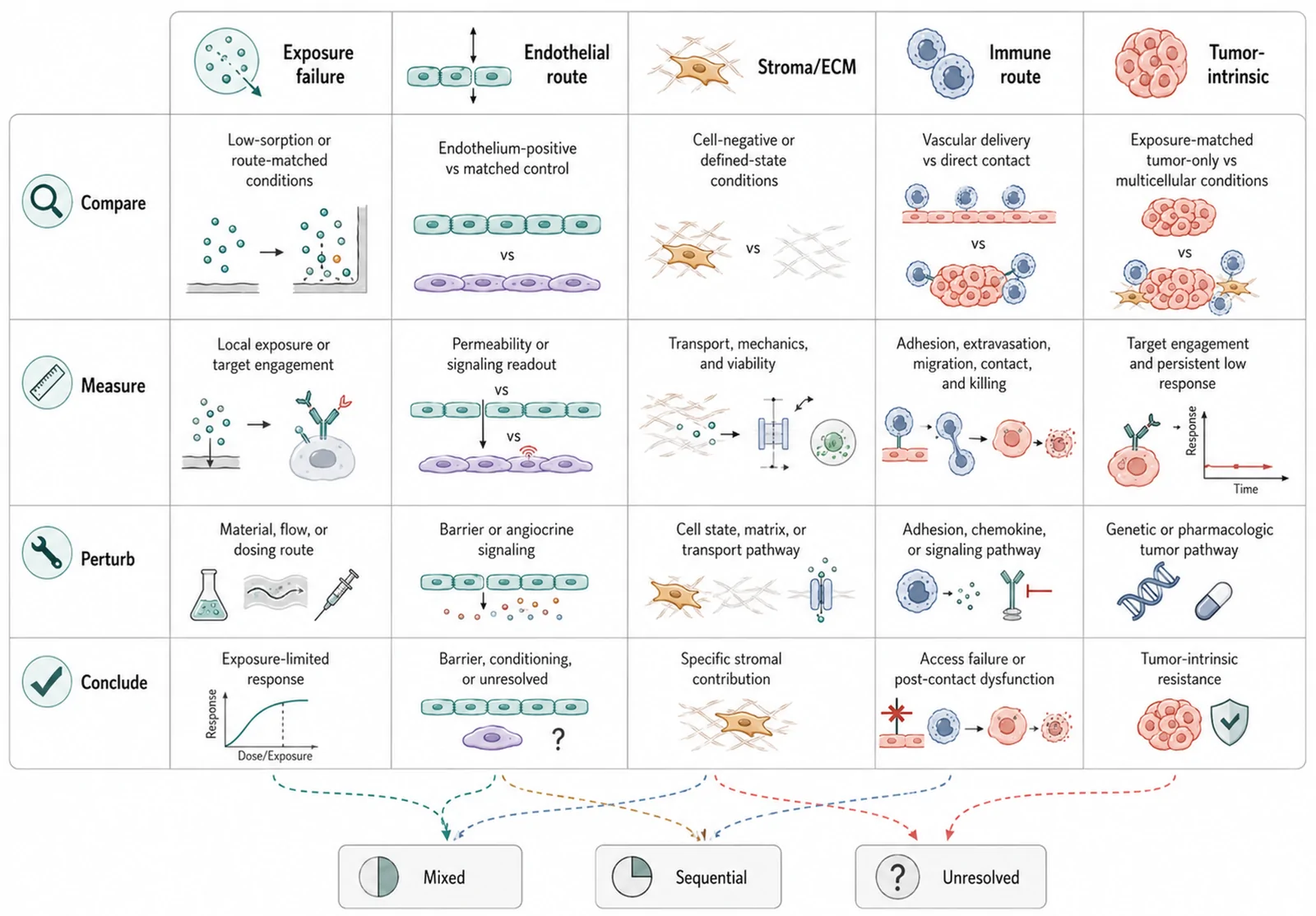
Mechanism–evidence map for five candidate routes to a weak treatment effect. Each mechanism is linked to the minimum comparison, measurement, and mechanism-directed perturbation needed for a mechanism-specific interpretation. Exposure failure requires local exposure or target-engagement evidence; endothelial mechanisms require separation of barrier and conditioning effects; stromal mechanisms require defined cell or matrix states; immune mechanisms require distinction between vascular access and post-contact function; and tumor-intrinsic resistance requires exposure matching and control of microenvironmental alternatives. Mixed, sequential, or unresolved interpretations are retained when alternatives remain coupled. ECM, extracellular matrix.

Figure 5 links each of the five candidate mechanisms to the minimum comparison, measurement, perturbation, and interpretation required. Table 3 provides representative study-level examples across Core and Boundary evidence, and Table 4 provides the corresponding evidence requirements and strongest defensible conclusions. When coupled alternatives cannot be separated, Section 5.6 retains a mixed, sequential, or unresolved interpretation.

### 5.1. Device- and Vascular-Level Exposure Failure

**Mechanistic premise.** Reduced efficacy after vascular-side dosing can result from compound loss in the device, inadequate lumen patency or perfusion, restricted transendothelial transfer, delayed matrix penetration, or an exposure history that differs from direct dosing [29,30,31,32,33,37,43,60]. Together, these possibilities constitute an exposure hypothesis that requires evaluation before biological resistance is assigned.

**Required evidence.** An exposure-limited interpretation requires a material- or circuit-recovery control, a relevant vascular-side concentration or validated delivery proxy, a geometry- and timing-matched direct-exposure comparator, and a tumor-compartment penetration or target-engagement measurement. A mechanism-directed manipulation of flow, route, patency, or barrier access must alter the proposed exposure step. Restoration of treatment response after effective exposure is increased provides the strongest support.

**Representative studies.** In Nashimoto et al. (Core), comparison of perfused with non-perfused conditions showed that perfusion altered tumor-spheroid growth and drug delivery [60]. Skubal et al. (Core) combined vessel-patency assessment with time-resolved solute-transport measurements under vascular-targeted conditions [43]. Together, these studies support a contribution of perfusion or vascular transport, while tracer behavior, flow-dependent cell state, and active local drug exposure remain partly unresolved.

**Supported interpretation.** With measured delivery and an appropriate comparator, the defensible conclusion is that inadequate device or vascular delivery contributed to reduced efficacy within the tested exposure window. Without active local exposure or target engagement, the result supports only a route-associated response difference, not intrinsic resistance. If weak response persists after exposure matching, stromal, immune, or tumor-intrinsic explanations remain.

### 5.2. Endothelial Barrier Versus Endothelial-Conditioned Response

**Mechanistic premise.** Endothelium can influence treatment through at least two experimentally separable routes: physical regulation of transendothelial transport and biological conditioning of tumor, stromal, or immune state [41,42,61,62]. The presence of endothelium alone therefore does not identify which route accounts for a response difference.

**Required evidence.** A barrier claim requires a geometry-matched endothelium-negative or permeability-modulated comparator together with permeability and tumor-compartment exposure or target engagement. A conditioning claim requires transport to be matched while endothelial secretory, inflammatory, metabolic, or angiocrine signaling is perturbed. Retrieval of tumor and endothelial compartments for molecular analysis can strengthen the distinction but cannot replace the exposure control.

**Representative studies.** Kim et al. (Core) linked endothelial context and vascular-side cisplatin exposure to viability and molecular readouts [61]. Ro et al. (Core) used an open three-dimensional platform to compare endothelial context and retrieve tissue for secretome and expression analyses [62]. Both studies support an endothelial contribution, but transport restriction and endothelial-conditioned cell state were not fully separated [61,62].

**Supported interpretation.** The evidence may support an endothelial barrier, endothelial conditioning, or an unresolved combination. Equivalent target engagement accompanied by different responses supports a conditioning route, whereas reduced tumor-compartment exposure supports a barrier route. A general conclusion that endothelium causes resistance is not supported unless these principal alternatives have been tested.

### 5.3. Perivascular-Cell, Fibroblast, and Extracellular-Matrix Protection

**Mechanistic premise.** Pericytes, defined fibroblast states, and the extracellular matrix can alter treatment response through distinct and sometimes opposing functions, including vascular stabilization, transport modification, mechanical compression, cytokine signaling, survival support, and immune remodeling [51,52,53,54,59,63,64,65,66,67,68]. These routes require separate evaluation rather than combination into a single undifferentiated stromal mechanism.

**Required evidence.** The minimum design uses a matched cell-negative or phenotypically defined cell-state comparator, verifies comparable effective drug exposure where an exposure-independent survival claim is made, and measures transport or mechanics separately from pathway activity and viability. A pathway-specific intervention or state conversion must reverse the proposed signaling or mechanical step without introducing a new exposure difference.

**Representative studies.** Maity et al. (Boundary) associated pericyte context with temozolomide response in a glioblastoma microphysiological model, although a definitive CCL5–CCR5 interpretation still requires pathway-specific reversal [54]. Farin et al. (Boundary) showed that matched patient-derived CAF states altered organoid therapy response without modeling vascular entry [63]. Offeddu et al. (Core) linked patient-specific mechanics, tissue compaction, pressure, and delivery in vascularized breast-desmoplasia models and included donor-related replication with orthogonal biomechanical and delivery readouts [64]. Nicolas et al. (Boundary) directly tested principal stromal alternatives through IL-1-, senescence-, and senolytic-directed interventions that connected inflammatory fibroblast state, matrix accumulation, and treatment resistance outside a vascularized chip [65].

**Supported interpretation.** The evidence supports a contribution of the specified perivascular cell, CAF state, matrix property, or pathway under the tested conditions; it does not support a general conclusion that all stroma is protective. Physical-barrier, survival-signaling, and vascular-remodeling routes remain separate claims. The contrasting effects of matrix modification and broad fibroblast depletion further require tumor type, stromal state, intervention, and endpoint to be stated explicitly [66,67,68].

### 5.4. Immune-Access Failure Versus Post-Contact Effector Dysfunction

**Mechanistic premise.** A terminal killing measurement integrates a sequence that includes endothelial adhesion, arrest, extravasation, stromal migration, tumor contact, target recognition, effector maintenance, and tumor sensitivity to inflammatory killing [44,45,55,56]. Failure at different steps can produce the same endpoint while implying different interventions [44,45,55,56].

**Required evidence.** The minimum design compares vascular delivery with direct tumor contact and measures viable immune-cell input, adhesion, extravasation, migration, tumor contact, antigen context, killing, cytokines, persistence, and immune-cell state at the relevant steps. The perturbation must match the failed step and may target adhesion, chemokine, endothelial, matrix, checkpoint, metabolic, or effector-signaling processes. Equivalent contact is required before post-contact dysfunction can be claimed.

**Representative studies.** Wisdom et al. (Core) isolated an endothelial access step by combining intercellular adhesion molecule 1 (ICAM-1) blockade with separate adhesion, extravasation, and migration readouts; the study distinguished a trafficking mechanism but did not establish tumor killing or persistence [44]. Bains et al. (Core) showed that vascular, macrophage, trafficking, and killing compartments could jointly contribute to CAR-T suppression, although access and post-contact effects remained partly coupled [45]. Dekkers et al. (Boundary) characterized engineered T-cell behavior after contact with patient cancer organoids [55]. Sun et al. (Boundary) included pharmacological and genetic reversal [56]. Interventions directed at programmed cell death protein 1 (PD-1) and TANK-binding kinase 1 (TBK1) restored post-contact CAR-T activity and altered both immune and tumor sensitivity, although vascular entry was absent from the model.

**Supported interpretation.** If direct-contact killing is preserved but vascular delivery fails, immune access is implicated. If trafficking and tumor contact are adequate but killing, cytokines, or persistence remain impaired, post-contact effector dysfunction is supported. When neither the access step nor equivalent contact has been measured, the result is limited to reduced immune efficacy with an unresolved failed step.

### 5.5. Tumor-Intrinsic Resistance After Exposure and Microenvironment Matching

**Mechanistic premise.** Tumor-intrinsic resistance is a residual mechanistic conclusion rather than a default explanation for reduced treatment response. It becomes testable only after the relevant therapy has reached the tumor compartment, engaged its target, and been evaluated against the vascular, endothelial, stromal, and immune alternatives relevant to the claim [8,56,63].

**Required evidence.** The minimum evidence chain includes active local exposure or target engagement, an exposure-matched tumor-only comparator, a multicellular condition that preserves the competing microenvironmental route, and a tumor-cell-directed genetic, pharmacological, or state perturbation. In immune studies, viable effector cells must reach and contact antigen-positive tumor cells before tumor-intrinsic resistance to immune killing is inferred.

**Representative studies.** Patient-derived organoids are valuable because they preserve interpatient heterogeneity and enable controlled exposure-response comparison [8,63]. Sun et al. further show that TBK1 targeting can increase tumor sensitivity to immune-mediated killing while also preventing CAR-T dysfunction, illustrating that a single intervention can act on both tumor-intrinsic and immune-cell routes [56]. These data support pathway contributions under controlled contact conditions but do not establish vascular delivery [56].

**Supported interpretation.** Intrinsic resistance is supported only when weak response persists despite adequate exposure, target engagement, and control of the relevant microenvironmental alternatives and when a tumor-cell-directed intervention modifies the phenotype. Without these conditions, the defensible wording is persistent low response after the measured controls, with intrinsic resistance remaining a candidate rather than a demonstrated mechanism.

### 5.6. Mixed, Sequential, and Unresolved Mechanisms

**Mechanistic premise.** Mechanisms can coexist or occur sequentially. Treatment can alter vascular permeability, induce fibroblast senescence, modify matrix deposition, impair immune-cell state, and select tumor-cell populations on different timescales [56,65]. Partial rescue of one step does not establish that the remaining response deficit arises from the same route [56,65].

**Required evidence.** Longitudinal and factorial designs are most informative when the mechanism is treatment-induced or time dependent. Measurements need to follow the proposed sequence, with prespecified early exposure or trafficking readouts, intermediate cell-state or matrix readouts, and later killing or viability outcomes. Sequential perturbations and compartment-specific rescue help determine whether routes are additive, dependent, or merely correlated.

**Representative studies.** Across the focused appraisal, sophisticated multicompartment models frequently included a comparator and a perturbation, but only a small subset directly tested the principal alternative. The resulting limitation was incomplete separation of coupled explanations, not insufficient model complexity. A minimal model with a matched comparator, measured exposure, and a specific perturbation can support a stronger mechanism claim than a highly complex model with only terminal viability.

**Supported interpretation.** When the available evidence supports more than one route without distinguishing among them, the interpretation remains mixed or unresolved. Figure 2 summarizes the overall evidence sequence and descriptive categories, Figure 5 maps the mechanism-specific requirements, Table 3 provides representative study-level examples, and Table 4 specifies the strongest defensible conclusion for each mechanism. Mechanistic attribution requires the compartments needed for the stated claim, not every possible compartment.

**Table 3:** Representative study-level examples across Core and Boundary evidence routes. The supported interpretation is limited to the reported comparator, perturbation, and readouts.

Study	Scope	Mechanistic Question	Comparator or Perturbation	Orthogonal Evidence	Supported Interpretation and Unresolved Alternative
Quintard et al. [40]	Boundary	Vascularized-organoid platform performance	Device and loading comparisons	Perfusion, growth, organoid function	Strong platform evidence; no tumor therapy-resistance mechanism was tested
Nashimoto et al. [60]	Core	Perfusion-dependent tumor delivery	Perfused versus static or non-perfused conditions	Perfusion, spatial delivery, tumor activity	Perfusion contributes; active local drug exposure and flow-dependent biology remain partly unresolved
Kim et al. [61]	Core	Endothelial context in cisplatin response	Endothelial context and vascular-side exposure	Viability and molecular profiling	Endothelium contributes; barrier and endothelial-conditioning routes remain partly unresolved
Maity et al. [54]	Boundary	Pericyte-associated temozolomide response	With versus without pericytes	Viability and CCL5-related molecular readouts	Pericyte context contributes; definitive pathway reversal and vascular transport are absent
Farin et al. [63]	Boundary	CAF-state-dependent therapy response	Organoid alone versus matched patient-derived stroma	Stromal-state profiling and drug response	Defined CAF state contributes; vascular entry is not modeled
Offeddu et al. [64]	Core	Biomechanical determinants of delivery	Patient-specific mechanical and desmoplastic comparisons	Pressure, compaction, spatial delivery	Biomechanics contributes to delivery differences; complete alternative exclusion and rescue are incomplete
Wisdom et al. [44]	Core	Endothelial control of T-cell entry	ICAM-1 blockade under defined chemokines	Adhesion, extravasation, migration	Endothelial adhesion mechanism distinguished for trafficking; killing and persistence are not established
Bains et al. [45]	Core	CAR-T suppression across compartments	Vascular and macrophage comparisons	Trafficking, immune state, tumor killing	Vascular and macrophage context contribute; access and post-contact effects remain partly coupled
Nicolas et al. [65]	Boundary	Treatment-induced inflammatory stromal resistance	IL-1, senescence, and senolytic interventions	CAF state, ECM, treatment response	Inflammatory stromal mechanism distinguished in boundary models; vascular entry remains untested
Sun et al. [56]	Boundary	Reversible post-contact CAR-T dysfunction	PD-1- and TBK1-directed interventions	Immune state, cytokines, killing, tumor sensitivity	Post-contact immune and tumor-sensitivity routes distinguished; vascular access is not modeled
Blackledge et al. [59]	Boundary	CAF-state effects on vascular and immune function	RestCAF versus ProCAF	Perfusability, ECM and secretome, monocyte recruitment	Defined CAF state contributes to vascular and recruitment differences; no tumor construct or treatment-rescue experiment

**Note.** This is a representative subset rather than the complete 25-study appraisal. Scope and evidence-component assignments are derived from Supplementary Tables S2A,B and S3A,B. Boundary evidence supports only the stated adjacent inference and is not equivalent to a vascularized tumor-model experiment. CAF, cancer-associated fibroblast; CAR-T, chimeric antigen receptor T cell; CCL5, C-C motif chemokine ligand 5; ECM, extracellular matrix; ICAM-1, intercellular adhesion molecule 1; IL-1, interleukin-1; PD-1, programmed cell death protein 1; TBK1, TANK-binding kinase 1.

**Table 4:** Mechanism-specific evidence requirements and strongest defensible conclusions.

Proposed Mechanism	Essential Comparator	Mechanism-Directed Perturbation	Required Orthogonal Measurement	Strongest Defensible Conclusion and Unresolved Alternative
Device/material exposure loss	Device recovery versus a lower-loss or material-free exposure condition	Material, coating, circuit, humidity, or recovery intervention	Administered, recovered, and local exposure or target engagement	Device loss contributes; endothelial, matrix, and intrinsic routes remain unless separately controlled
Vascular perfusion or delivery failure	Vascular-side delivery versus exposure-matched direct delivery	Flow, route, lumen patency, or vascular-access manipulation	Perfused fraction plus local concentration or penetration and target engagement	Inadequate vascular delivery contributes within the measured exposure window
Endothelial barrier	Endothelium-positive versus geometry-matched barrier control	Junctional or permeability modulation	Permeability together with tumor-compartment exposure or engagement	The endothelial barrier contributes; endothelial conditioning remains unless transport is matched
Endothelial conditioning	Signaling-separated condition with transport matched	Angiocrine, inflammatory, or metabolic pathway perturbation	Endothelial and tumor-cell state plus response at equivalent exposure	Endothelial signaling contributes independently of the measured transport difference
Perivascular-cell protection	Perivascular-cell-positive versus matched negative or defined state	Cell-state or candidate-pathway perturbation	Exposure, tumor survival, and pathway activity measured separately	The tested perivascular state contributes; vascular stabilization and signaling may remain coupled
CAF or ECM protection	Stromal-negative or defined CAF/matrix-state comparison	CAF-state, matrix, cytokine, or mechanotransduction perturbation	Transport and mechanics measured separately from survival signaling	The specified stromal route contributes; the result is not generalized to all CAFs or matrices
Immune-access failure	Vascular delivery versus direct tumor contact	Adhesion, chemokine, endothelial, or matrix perturbation	Adhesion, extravasation, migration, tumor contact, and viable cell input	Access limits efficacy before target engagement; post-contact competence remains to be tested
Post-contact effector dysfunction	Equivalent tumor contact and antigen context	Checkpoint, metabolic, or effector-signaling perturbation	Killing, cytokines, persistence, and immune-cell state	Post-contact dysfunction persists despite adequate access; tumor sensitivity may remain a competing route
Tumor-intrinsic resistance	Exposure-matched tumor-only and multicellular conditions	Tumor target or resistance-pathway perturbation	Active exposure, target engagement, and tumor-cell state	Intrinsic resistance is supported after relevant transport and microenvironmental alternatives are controlled
Mixed or sequential mechanisms	Factorial conditions that isolate the proposed sequence	Time- or compartment-specific perturbations and partial rescues	Longitudinal exposure, state, trafficking, and response readouts	More than one route contributes, or the failed step remains unresolved; no exclusive mechanism is claimed

**Note.** These requirements synthesize established principles of mechanistic reasoning and the study-level practices identified in the focused appraisal [25,26,27]. The table is an interpretive aid rather than a risk-of-bias instrument, regulatory qualification system, or clinical decision rule. Rescue or reversal provides additional evidence of mechanism dependence but may be infeasible in some technical or biological settings. CAF, cancer-associated fibroblast; ECM, extracellular matrix.

The mechanism-specific requirements also provide the basis for interpreting apparently contradictory findings. The same intervention can alter transport, cell state, and treatment response in different directions when the model boundary, treatment window, or endpoint changes.

## 6. Sources of Heterogeneity in Mechanistic Interpretation

**Figure 6:**
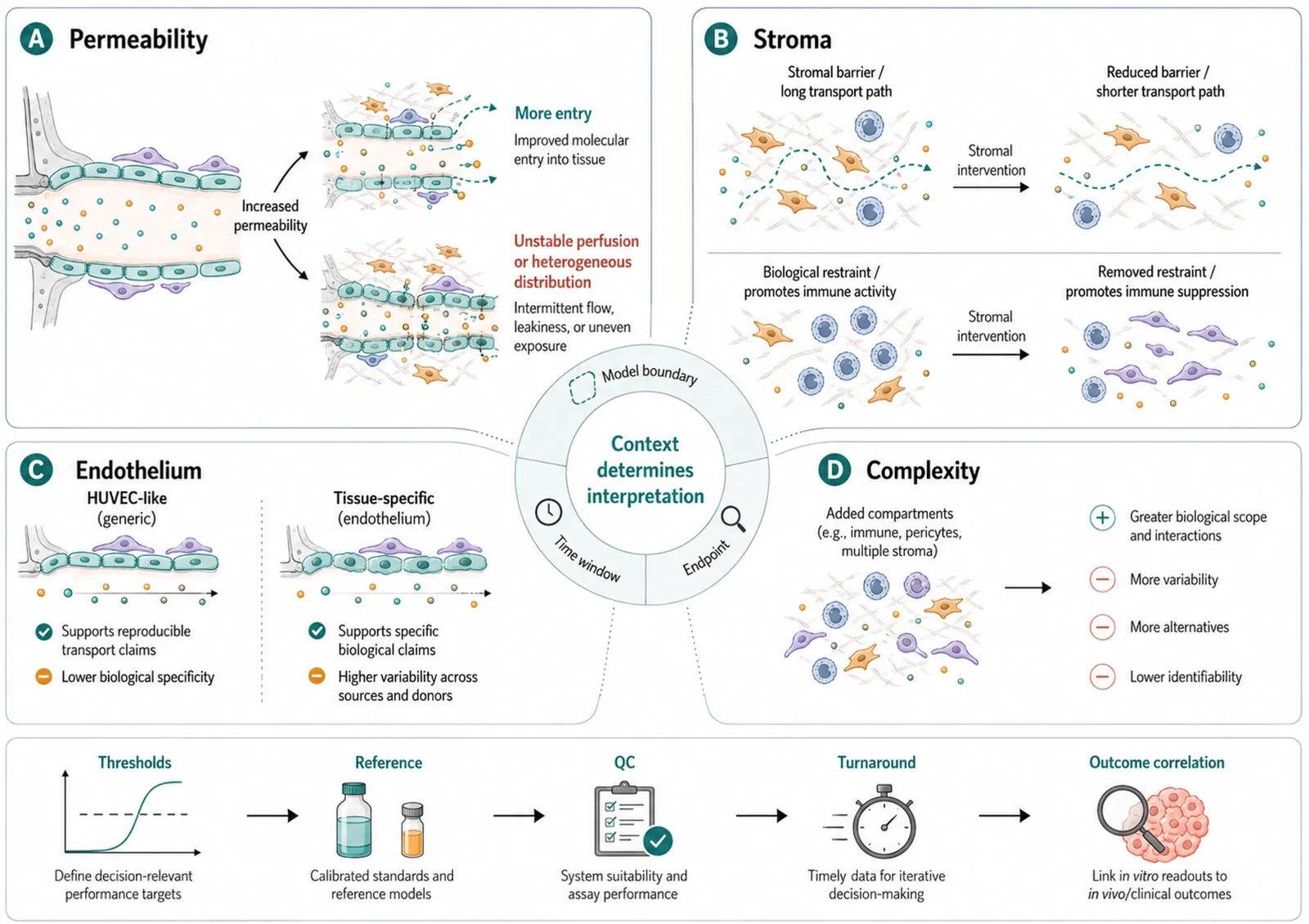
Context-dependent trade-offs that can produce apparently discordant findings. Increased permeability can improve molecular entry while destabilizing perfusion or producing heterogeneous distribution; stromal intervention can shorten a transport path while removing biological restraint or altering immune regulation; generic and tissue-specific endothelium support different transport and signaling claims; and additional compartments increase both biological scope and competing explanations. Model boundary, treatment window, and endpoint determine the defensible interpretation. Progress toward a fit-for-purpose application additionally requires prespecified thresholds, reference treatments, quality-control criteria, acceptable turnaround, and association with relevant outcomes. HUVEC, human umbilical vein endothelial cell; QC, quality control.

Figure 6 summarizes four recurrent sources of heterogeneity in the interpretation of vascularized tumor-model evidence. Across these examples, the direction and mechanistic meaning of an observed effect depend on the model boundary, treatment window, therapeutic route, and selected endpoint.

### 6.1. Permeability, Perfusion, and Spatial Drug Distribution

Increased permeability can improve molecular entry while coinciding with unstable perfusion, elevated interstitial pressure, or heterogeneous distribution [2,37,43,64]. Conversely, vascular normalization can improve delivery within a treatment window, whereas excessive vascular suppression can reduce perfusion [2,5]. A conclusion about improved delivery requires time-resolved measurement of both vascular transport and intratumoral exposure rather than inference from vessel leakage or morphology alone [2,5,37,43,64].

### 6.2. Stromal Modulation and Context-Dependent Treatment Effects

The pancreatic-cancer literature shows that reducing fibroblastic stroma can produce adverse effects even when a physical barrier is diminished [66,67,68]. Vascular microphysiological systems further demonstrate that CAF states differ in their capacity to support perfusable vessels and recruit immune cells [59]. Interpretation of a stromal intervention depends on the defined cell state, transport effect, signaling pathway, and therapeutic endpoint rather than on a uniform increase or decrease in “stroma.”

### 6.3. Endothelial Provenance and Inference Specificity

HUVEC-based systems are useful for controlled transport and trafficking studies, but they do not reproduce every organ-specific endothelial program [37,38,41]. Tissue-specific endothelial cells can increase biological specificity while introducing donor- and culture-associated variation [41]. These model classes support different inferences rather than a simple validity hierarchy: generic endothelium can support a measured transport or trafficking claim, whereas organ-specific signaling requires appropriate cell identity and phenotypic validation [37,38,41].

### 6.4. Model Complexity, Interpretability, and Decision Relevance

Additional compartments can reveal clinically relevant interactions, but they also introduce new sources of variation and additional routes to the same endpoint. Model composition, mechanism-attribution evidence, analytical reproducibility, and intended-use validation are therefore separate dimensions of platform evaluation. Because no common head-to-head benchmark exists and performance depends on the intended use, a universal ranking of vascularized tumor-model technologies would be misleading. Platforms are more appropriately compared according to whether they resolve the biological step and performance requirements relevant to a defined decision.

## 7. Translational Applications and Fit-for-Purpose Validation

### 7.1. Mechanism-Informed Applications in Translational Drug Development

The near-term translational value of vascularized tumor models lies in reducing uncertainty during translational drug development. These systems can help determine whether a candidate intervention is limited by vascular delivery, endothelial state, stromal protection, immune access, post-contact dysfunction, or a coupled process, thereby informing compound prioritization, combination hypotheses, and confirmatory study design [21,22,23,24,46,47,48,49,69]. The available evidence does not support autonomous patient-level therapy selection. Mechanism-informed screening currently functions as an intermediate step between experimental biology and a prespecified clinical or drug-development question.

### 7.2. Clinical Context of Use and Fit-for-Purpose Validation

Clinical translation begins with a defined context of use: the specific purpose, population, model boundary, and decision that the platform is intended to inform [47,48,49,69]. Potential contexts include mechanistic prioritization of candidate therapies, selection of combinations for further testing, enrichment of clinical-trial hypotheses, or prospective prediction of treatment response. Each context requires evidence at four levels. Analytical performance includes repeatability, reproducibility, failure rate, reference controls, and stable thresholds. Biological validity requires relevant exposure, target engagement, and a response mechanism that is appropriate to the intended decision. Clinical validity requires independent and preferably prospective association with patient outcomes. Clinical utility requires evidence that use of the assay improves a decision relative to the current standard. The required extent of validation depends on the consequence of the intended use [46,47,48,49,69].

### 7.3. Minimum Reporting Requirements for Translational Evaluation

A translational report needs to state the context of use and candidate mechanism, describe the engineering and biological boundary conditions, and report the therapeutic route and local exposure. It also needs a bottleneck-matched comparator, a mechanism-directed perturbation, an orthogonal or step-resolved functional measurement, disclosure of technical failures and replication, and identification of the principal alternatives not excluded. When image analysis or machine learning is used, reporting needs to cover acquisition, reference labels, feature definitions, and validation against biological function [57,58]. Plate-compatible formats, closed fluidics, low-sorption materials, automated loading, and standardized analysis workflows can reduce selected sources of pre-analytical variation [40,46]; however, technical scalability does not substitute for biological or clinical validation.

## 8. Methodological Limitations and Priorities for Clinical Validation

The focused appraisal prioritized mechanistically informative experimental designs. Consequently, the findings do not estimate field-wide platform prevalence or performance. Heterogeneity in device architecture, biological composition, treatment modality, dosing route, and outcome definition precluded quantitative synthesis and limits direct cross-platform comparison. Incomplete reporting of local exposure, target engagement, technical failure rates, and independent replication further constrains assessment of mechanism attribution. Clinical translation cannot yet be assessed because the included studies did not provide cross-site or prospective validation against prespecified performance thresholds and patient outcomes. These limitations identify the principal next steps for translation: standardized reference conditions, prespecified acceptance criteria, multicenter reproducibility, and prospective evaluation of clinical validity and utility.

## 9. Conclusions and Clinical Translation Priorities

A mechanism-specific interpretation in a vascularized tumor model is justified when the experiment includes a comparator that differs in the proposed bottleneck and measures local exposure or the relevant biological step. It also requires perturbation of the proposed mechanism and confirmation of an independent functional consequence. Direct testing of the principal competing explanation strengthens discrimination, and rescue or reversal provides additional evidence that the phenotype depends on the proposed mechanism. When these conditions are not met, the interpretation remains contributory, mixed, or unresolved rather than assigning an exclusive resistance mechanism.

The focused appraisal shows that most studies can support mechanism-informed screening or experimental prioritization, whereas evidence for clinical validation and utility remains limited. Twenty-one of 25 studies combined a relevant comparator, mechanism-directed perturbation, and functional readout; four also tested the principal competing explanation, and three included rescue or reversal. Fourteen reported a reproducibility-oriented feature or independent benchmark, but none reported cross-site, prospective, threshold-based, or comparable fit-for-purpose validation. These findings identify the next experiments required to connect mechanistic interpretation with decision relevance.

Progress toward clinical use depends less on adding compartments than on defining a context of use and demonstrating analytical performance, biological validity, clinical validity, and, ultimately, clinical utility. Exposure-limited response requires measurement of local exposure or target engagement; endothelial, stromal, and immune mechanisms require separation of the relevant sequential steps; and tumor-intrinsic resistance requires adequate exposure and control of microenvironmental alternatives. Prospective validation against patient outcomes is required before these models can support treatment selection. Aligning mechanistic evidence with that clinical validation pathway can make vascularized tumor organoid and tumor-on-chip systems more useful for therapy development and precision oncology.

## Figures and Tables

**Table 1:** Selected recent reviews and their organizing emphases relevant to mechanism attribution.

Review	Primary Organizing Emphasis	Principal Contribution	Relevance to Mechanism Attribution
Konopka et al. (2024) [19]	Vascularized tumor-on-chip models and neovasculature	Surveys platform architectures and applications for vascular and treatment studies	Provides the platform context for identifying which vascular function or treatment step is experimentally resolved
Kim et al. (2025) [20]	Tumor–microenvironment–drug interactions in vascularized chips	Integrates vascularized models with microenvironmental and pharmacological applications	Provides interaction-level context for distinguishing delivery effects from multicellular response
Poljak et al. (2026) [21]	Tumor vascular biology, perfusion engineering, and therapy innovation	Connects pathological vasculature with device engineering and therapeutic opportunities	Provides the engineering and translational context for bottleneck-specific experimental tests
Menzani et al. (2026) [22]	Strategies for vascularizing organoids on chip	Compares top-down, bottom-up, and hybrid approaches, functional performance, and personalization	Provides the design context for evaluating how vascularization choices constrain interpretation
Wu et al. (2026) [23]	Cellular and molecular regulation of tumor-organoid vasculature	Links angiogenic regulation and vascular function to targeted therapy and precision oncology	Provides biological hypotheses that require experiment-level discrimination from transport and intrinsic-response alternatives
Huang et al. (2026) [24]	Vascular transport, endothelial state, drug exposure, and therapeutic interpretation	Frames treatment response as a composite of transport, exposure, endothelial behavior, and intrinsic sensitivity	Clarifies the transport, exposure, endothelial, and intrinsic-response variables that must be separated in mechanism attribution
Current review	Study-level requirements for distinguishing competing therapy-response mechanisms	Defines mechanism-specific evidence sets and applies them to 25 eligible primary studies	Identifies whether an experiment supports association, contribution, or discrimination and specifies the validation needed for context-specific translational use

**Table 2:** Evidence-supported boundary conditions, associated risks of mechanistic misinterpretation, and minimum controls.

Boundary Condition	Evidence-Supported Effect	Risk of Mechanistic Misinterpretation	Minimum Control/Reporting Requirement	Strongest Conclusion Without the Control
PDMS sorption or evaporation	Reduced small-molecule recovery or osmolality shift	Device-associated loss misattributed to endothelial or tumor resistance	Material-only recovery, humidity/volume control, and local exposure proxy	Only nominal-dose response can be reported
Geometry, bonding, or interface variability	Changes in channel dimensions, pressure drop, leakage, or delamination	Manufacturing variation misattributed to a biological or treatment effect	Critical dimensions and tolerances, bonding method, batch identifier, leak test, and interface acceptance criteria	Batch-specific feasibility only
Bubble, interrupted flow, leakage, or occlusion	Shear spike, loss of patency, or heterogeneous exposure	Device failure misattributed to biological non-response	Priming record, patency or connected perfusion measure, exclusions, and failure denominator	The run cannot support mechanism attribution
Direct versus vascular-side dosing	Different concentration-time histories across endothelium and matrix	Delivery failure misattributed to tumor-intrinsic insensitivity	Route-matched timing plus tissue exposure or target-engagement measurement	Only route-associated response differences are supported
Patterned lumen versus self-assembled network	Defined geometry versus variable connectivity and diameter	Network failure misattributed to an endothelial barrier effect	Perfused fraction, permeability, stability, and chip-level variability	Architecture-specific feasibility, not a general vascular mechanism
HUVEC versus tissue-specific endothelium	Different permeability and endothelial phenotype	Generic barrier findings generalized to organ-specific endothelial biology	Cell source, phenotype, permeability, and claim restriction	Generic transport claim only
Natural versus defined matrix	Different biochemical definition, mechanics, degradability, and transport	Matrix-associated response interpreted as a defined transport or signaling mechanism without measuring the relevant matrix property	Composition and lot, modulus, degradation, ligand and transport metrics	Matrix association, not a defined transport or signaling mechanism
With versus without mural cells or CAF states	Barrier stabilization, remodeling, and paracrine survival can coexist	A cell-state-specific effect generalized to all stromal cells	Matched cell-negative/state comparison plus transport, mechanics, and signaling readouts	Contribution of the tested cell state only
Immune-cell product and antigen context	Product source, engineering state, donor matching, and antigen context can alter access and killing readouts	Product- or donor-specific immune behavior generalized across contexts	Donor/product identity, HLA/antigen context, activation, engineering, viability, and reference control	Product- and context-specific inference only

**Note.** The third column identifies the specific mechanistic misinterpretation that may arise when the corresponding boundary condition is not measured or controlled. The listed effects are conditional on the material, device, and operating configuration; they are not universal failure modes. The final column states the strongest conclusion supportable without the indicated control or reporting requirement. CAF, cancer-associated fibroblast; HLA, human leukocyte antigen; HUVEC, human umbilical vein endothelial cell; PDMS, polydimethylsiloxane.

## Data Availability

All data supporting this Review were derived from published literature. Retained source records and the corresponding study-level extraction and experimental-design-feature data are provided in Supplementary Tables S2A,B and S3A,B. No new experimental or clinical dataset was generated.

## Supplementary Materials

The following supporting information can be downloaded at: https://www.mdpi.com/article/10.3390/bioengineering13080947/s1, Supplementary Figure S1: Scope-classification decision tree for Core and Boundary evidence; Supplementary Figure S2: Published mechanism-focused image–function pairs; Supplementary Tables S1A,B: Search strategy, information sources, and study-selection rules; Supplementary Tables S2A,B: Study-level model, comparator, perturbation, exposure, orthogonal-readout, rescue, competing-alternative, and interpretation fields; Supplementary Tables S3A,B: Experimental-design-feature definitions, counts, and study assignments. Primary studies represented in Supplementary Tables S2A–S3B that are not otherwise cited in the main synthesis are included in the main reference list and cited here [70,71,72,73,74,75,76,77,78,79,80]. The published source panels used in Supplementary Figure S2 are likewise included in the main reference list and cited here [81,82,83,84].

## Abbreviations

CAF, cancer-associated fibroblast; CAR-T, chimeric antigen receptor T-cell; EC, endothelial cell; ECM, extracellular matrix; HLA, human leukocyte antigen; HUVEC, human umbilical vein endothelial cell; ICAM-1, intercellular adhesion molecule 1; PD-1, programmed cell death protein 1; PDMS, polydimethylsiloxane; TBK1, TANK-binding kinase 1.
