# Supplementary material for: Functional Vascularization in Tumor Organoid and Tumor-on-Chip Models: Evidence Requirements for Attributing Therapy-Resistance Mechanisms
Source: Bioengineering (Basel). 2026 Aug 21;13(8):947. doi: 10.3390/bioengineering13080947 (PMC13509796; doi:10.3390/bioengineering13080947)
Supplement: Supplementary file 1 [file bioengineering-13-00947-s001.zip › bioengineering-4446161-supplementary.pdf]

Supplementary Materials: Functional Vascularization in Tumor Organoid and Tumor-on-Chip Models: Evidence Requirements for Attributing Therapy-Resistance Mechanisms

Yafang Li, Yiyi Yang, Lina Zhao, Zihao Bian and Zongjiu Zhang*

Department of Biomedical Engineering, Tsinghua University, Beijing 100084, China

Supplementary Materials

This supplement reports the search strategy and study-selection rules (S1A-S1B), study-level extraction and supported interpretations (S2A-S2B), experimental-design-feature definitions and assignments (S3A-S3B), a scope-classification decision tree (Supplementary Figure S1), and mechanism-focused published image-function pairs (Supplementary Figure S2). The 25-study set was assembled for mechanistic appraisal; its component counts do not estimate prevalence in the wider field.

Abbreviations: CAF, cancer-associated fibroblast; CAR-T, chimeric antigen receptor T-cell; ECM, extracellular matrix; ICAM-1, intercellular adhesion molecule 1; MPS, microphysiological system; PD-1, programmed cell death protein 1; TBK1, TANK-binding kinase 1.

Supplementary Table S1A. Search strategy and information sources.

Database or source	Search date	Search role	Search concept	Complete or representative search string
PubMed	2026-07-31	Database search	Vascularized tumor models and therapy mechanisms	((vascularized[Title/Abstract] OR perfused[Title/Abstract] OR endothelial[Title/Abstract]) AND (tumor-on-chip[Title/Abstract] OR cancer-on-chip[Title/Abstract] OR tumor organoid-on-chip[Title/Abstract] OR vascularized tumor organoid[Title/Abstract] OR microphysiological system[Title/Abstract])) AND (drug delivery[Title/Abstract] OR therapy response[Title/Abstract] OR resistance[Title/Abstract] OR immunotherapy[Title/Abstract] OR T-cell trafficking[Title/Abstract] OR CAR-T[Title/Abstract])
Web of Science	2026-07-01	Database search	Vascularized tumor models and therapy mechanisms	TS=((vascularized OR perfused OR endothelial) NEAR/3 (tumor-on-chip OR cancer-on-chip OR tumor organoid-on-chip OR vascularized tumor organoid OR microphysiological system)) AND TS=(drug delivery OR therapy response OR resistance OR immunotherapy OR T-cell trafficking OR CAR-T)
Scopus	2026-07-01	Database search	Vascularized tumor models and therapy mechanisms	TITLE-ABS-KEY((vascularized OR perfused OR endothelial) W/3 (tumor-on-chip OR cancer-on-chip OR tumor organoid-on-chip OR vascularized tumor organoid OR microphysiological system)) AND TITLE-ABS-KEY(drug delivery OR therapy response OR resistance OR immunotherapy OR T-cell trafficking OR CAR-T)
Google Scholar	2026-07-31	Database search	Targeted review and primary-study retrieval	"vascularized tumor-on-chip" therapy resistance; "vascularized tumor organoid" therapeutic response; "tumor microphysiological system" endothelial barrier drug; "vascularized tumor" CAR-T extravasation
Publisher records and citation tracking	2026-07-31	Supplementary source retrieval	Recent reviews, bibliographic verification, and mechanism-informative primary studies	Backward and forward citation tracking from the six overlapping reviews summarized in Table 1 and mechanistically informative primary studies
All information sources	2026-07-31	Mechanism-oriented retrieval	Mechanism-discriminating design terms	matched comparator OR exposure-matched OR mechanism-specific perturbation OR rescue OR reversal OR target engagement OR orthogonal readout combined with vascularized tumor-chip terms

Supplementary Table S1B. Focused-appraisal assembly and study-selection rules.

Method element	Selection rule	Interpretive boundary
Review purpose	Structured literature search, narrative synthesis, and focused qualitative evidence-component appraisal.	No pooled effect or field-wide prevalence estimate was attempted.
Information sources	PubMed, Web of Science, Scopus, and Google Scholar searches through 31 July 2026, supplemented by publisher records and backward/forward citation tracking.	Search strings and dates are reported in Supplementary Table S1A.
Unit of analysis	Distinct primary experimental study.	Online-first/final records and corrections linked to the same parent study; correction not counted separately.
Basic eligibility	Original experimental content, verifiable bibliographic identity, and direct relevance to a prespecified mechanism or necessary vascular-platform principle.	Required before Core/Boundary classification.
Core route	Three-dimensional tumor construct plus integrated vascular/endothelial interface plus mechanism-relevant functional endpoint.	Direct vascularized tumor-organoid or tumor-on-chip evidence.
Boundary route	Direct adjacent mechanistic or essential platform evidence lacking at least one Core criterion.	Retained only for the explicitly bounded inference.
Exclusion route	Review, perspective, duplicate report, unverifiable record, or experimental report too remote from the prespecified mechanisms.	Not entered into the focused appraisal.
Selection sequence	First verify eligibility and study identity; second assign Core or Boundary under the fixed rule.	Selection and scope are separated from the qualitative evidence-component appraisal.
Missing information	Unreported or indeterminate fields retained as not reported or unclear.	Missing fields do not satisfy an evidence component.
Final focused set	25 studies: 18 Core and seven Boundary.	Focused mechanistic sample; not an exhaustive census of the field.

Supplementary Table S2A. Focused study-level extraction: model scope and mechanistic question.

Study	Scope	Primary mechanistic role	Matched comparator	Mechanism-specific perturbation	Replication/QC evidence	Primary source
Agarwal et al. (2017)	Core	Vascular architecture and drug response	Vascularized versus related non-vascularized/control constructs	Anticancer drug treatment; no isolated vascular mechanism intervention	No explicit multi-donor, batch, QC, or independent-validation evidence identified	10.1021/acsnano.7b00824
Shirure et al. (2018)	Core	Perfused vascular delivery and drug-sensitivity testing	Cell-line and patient-derived organoid conditions with perfused platform comparisons	Drug treatment and platform-condition comparisons; no isolated bottleneck perturbation	Repeated testing across cell-line and patient-derived models; independent model types reported	10.1039/C8LC00596F
Nashimoto et al. (2020)	Core	Perfusion-dependent delivery	Perfused versus non-perfused/static conditions	Flow/perfusion status manipulated	No explicit multi-donor, batch, QC, or independent-validation evidence identified	10.1016/j.biomaterials.2019.119547
Hachey et al. (2021)	Core	Reproduction of standard-of-care response	Treatment controls and comparison with in vivo response patterns	Standard treatment comparison, not a mechanism-specific bottleneck intervention	Independent in vivo benchmark and more than one colorectal model condition reported	10.1039/D0LC01216E

Continued on next page

Supplementary Table S2A (continued)

Study	Scope	Primary mechanistic role	Matched comparator	Mechanism-specific perturbation	Replication/QC evidence	Primary source
Hu et al. (2022)	Core	Vascular protection combined with chemotherapy	Single-treatment versus combination-treatment conditions	DMOG-related vascular intervention with paclitaxel or cisplatin	No explicit multi-donor, batch, QC, or independent-validation evidence identified	10.1021/acsbiomaterials.1c01099
Nicolas et al. (2022)	Boundary	Treatment-induced inflammatory stromal resistance	CAF states and treatment/intervention controls	IL-1 inhibition, senescence prevention, and senolytic intervention	Multiple experimental systems with patient-associated evidence and independent in vivo/clinical context	10.1016/j.ccell.2022.01.004
Hachey et al. (2023)	Core	Patient-specific vascularized treatment response and TGF-β-related contribution	FOLFOX, TGF-β receptor 1 inhibitor, and combination conditions across patient-derived models	TGF-β receptor 1 inhibition with standard therapy	Six patient samples reported with repeated patient-specific testing	10.1016/j.trsl.2022.11.011
Kim et al. (2023)	Core	Endothelial barrier versus endothelial-conditioned response	With versus without endothelium; sensitive versus resistant cells	Endothelial context plus vascular-side cisplatin exposure	No explicit multi-donor, batch, QC, or independent-validation evidence identified	10.1088/1758-5090/acef99
Wisdom et al. (2023)	Core	Endothelial control of T-cell adhesion and migration	Defined chemokine conditions and ICAM-1 blockade controls	ICAM-1 blockade and chemokine modulation	No explicit multi-donor, batch, QC, or independent-validation evidence identified	10.14573/altex.2208121
Farin et al. (2023)	Boundary	CAF-state-dependent therapy response	Organoid alone versus matched patient-derived CAF coculture	Stromal inclusion and chemogenomic/pathway perturbations	Patient biobank with multiple matched cases	10.1158/2159-8290.CD-23-0050
Dekkers et al. (2023)	Boundary	Post-contact engineered T-cell behavior	Engineered-cell product and target-context comparisons	Product and target-context manipulations	Large-scale tracking across patient-derived organoids and engineered-cell conditions	10.1038/s41587-022-01397-w
Offeddu et al. (2024)	Core	Biomechanical limitation of intratumoral delivery	Patient-specific mechanical/desmoplastic conditions and treatment comparisons	Matrix/tissue-mechanics comparisons and modulation	Patient-specific models with repeated donor-related assessments	10.1002/advs.202402757
Quintard et al. (2024)	Boundary	Reproducible vascularized-organoid platform performance	Device, loading, and culture-condition comparisons	Device and loading manipulations; no therapy-resistance mechanism perturbation	Parallelized platform across multiple organoid/tissue types with documented functional testing	10.1038/s41467-024-45710-4

Continued on next page

Supplementary Table S2A (continued)

Study	Scope	Primary mechanistic role	Matched comparator	Mechanism-specific perturbation	Replication/QC evidence	Primary source
Du et al. (2025)	Core	Tumor-specific vascularization, metastasis, and anti-vascular targeting	Vascularized PDTO conditions, tumor-vascular comparisons, and treatment controls	Notch/anti-vascular or pathway-directed perturbations reported	Patient-derived organoid platform with repeated personalized assessments reported	10.1002/adma.202412815
Skubal et al. (2025)	Core	Tumor neovascularizatio n, perfusion, and vascular-targeted therapy	Flow/perfusion and vascular-targeted treatment conditions	Vascular-targeted perturbation with flow-related comparison	No explicit multi-donor, batch, QC, or independent-validation evidence identified	10.7150/thno.95334
Maity et al. (2025)	Bound ary	Pericyte-associated temozolomide resistance	With versus without pericytes under temozolomide	Pericyte coculture and CCL5-CCR5 pathway analysis	No explicit multi-donor, batch, QC, or independent-validation evidence identified	10.1016/j.actbio.2025.05.038
Bains et al. (2025)	Core	Vascular and macrophage-mediated suppression of CAR-T cells	Compartment and macrophage-state comparisons	Macrophage-state and immunosuppressi ve-condition manipulations	No explicit multi-donor, batch, QC, or independent-validation evidence identified	10.1039/D4LC01089B
Liu et al. (2025)	Core	CAR-T vascular access, activity, and pharmacologicall y reversible dysfunction	Control CAR-T products, chemokine-directed constructs, treatment conditions, and matched tumor models	Chemokine engineering and pharmacological target modulation	Multiple human tumor constructs with independent in vivo validation and repeated experimental conditions	10.1038/s41587-025-02845-z
Ro et al. (2025)	Core	Endothelial interaction and treatment response	Endothelial context and candidate-target treatment comparisons	Endothelial context and candidate-target perturbation	Large spheroid numbers demonstrate throughput, but explicit biological multi-donor/batch/QC or independent validation was not confirmed	10.1002/advs.202410659
Bang et al. (2025)	Core	Angiogenic sprouting and vascular-targeted treatment response	Untreated, 5-FU, and sunitinib conditions in patient-derived spheroids	Sunitinib as a vascular-directed perturbation with chemotherapy comparison	Patient-derived tumor spheroids from head-and-neck cancer patients; multiple patient samples indicated	10.1007/s10544-025-00748-z
Shin et al. (2025)	Core	Tumor-angiogenesis interaction and anti-vascular drug response	Tumor/endothelia l spheroid configurations and treatment controls	Bevacizumab or other angiogenesis-directed treatment with drug-response comparison	Multiple tumor contexts and independent in vivo/model comparison reported	10.1016/j.mtbio.2025.101741

Continued on next page

Supplementary Table S2A (continued)

Study	Scope	Primary mechanistic role	Matched comparator	Mechanism-specific perturbation	Replication/QC evidence	Primary source
Blackledge et al. (2026)	Boundary	CAF-state-dependent vascular and immune function	RestCAF versus ProCAF conditions	Defined CAF-state comparison and related perturbations	Multiple CAF states; explicit donor/batch/QC evidence not sufficient by itself to establish reproducibility	10.1007/s10456-026-10061-9
Ruan et al. (2026)	Core	Vascularized cervical cancer organoid response and anti-VEGF treatment	Cisplatin, bevacizumab, and treatment-control conditions	Anti-VEGF perturbation and chemotherapy comparison	No explicit multi-donor, batch, QC, or independent-validation evidence confirmed	10.1016/j.canlet.2025.218110
Dai et al. (2026)	Core	Patient-specific vascularized colorectal tumoroid drug testing	Treatment conditions including FOLFOX and bevacizumab with patient-derived comparisons	Chemotherapy and anti-VEGF perturbations	Platform validation across 22 analyzed samples and repeated patient-derived testing	10.1016/j.xcrm.2026.102873
Sun et al. (2025)	Boundary	Reversible post-contact CAR-T dysfunction	Control, PD-1-directed, and TBK1-directed conditions in patient-derived spheroids	PD-1 blockade and TBK1 inhibition/deletion	Multiple patient-derived organotypic spheroids with pharmacological and genetic validation	PMID 39785827

Supplementary Table S2B. Focused study-level extraction: discriminating evidence and supported interpretation.

Study	Effective/local exposure and target engagement	Orthogonal readouts	Rescue/reversal	Principal alternatives tested	Supported interpretation
Agarwal et al. (2017)	Exposure: Drug delivery/penetration reported; exact local concentration not established; Target engagement: No	Perfusion/network imaging; Tumor growth or viability	No	Device, matrix, and exposure effects not independently separated	Findings are consistent with vascular architecture and drug response, but the causal route is not isolated.
Shirure et al. (2018)	Exposure: Vascular-side delivery and permeability/transport assessed; active local exposure not fully matched; Target engagement: No	Perfusion and vascular morphology; Tumor growth and drug response	No	Transport and tumor-intrinsic effects remain incompletely separated	Findings are consistent with perfused vascular delivery and drug-sensitivity testing, but the causal route is not isolated.
Nashimoto et al. (2020)	Exposure: Spatial drug-delivery readout; active local concentration/target engagement not established; Target engagement: No	Perfusion and vessel imaging; Spatial delivery plus spheroid growth/response	No	Flow, oxygenation, and delivery change together	Perfusion-dependent delivery contributes under the tested conditions; important competing explanations remain.
Hachey et al. (2021)	Exposure: Therapy delivered through vascularized model; effective local exposure not independently measured; Target engagement: No	Vascularized microtumor imaging; Treatment-response benchmark against in vivo patterns	No	Vascular, stromal, and tumor-intrinsic contributions are not separated	Findings are consistent with reproduction of standard-of-care response, but the causal route is not isolated.
Hu et al. (2022)	Exposure: No direct active local drug concentration or target-engagement measurement identified; Target engagement: No	Vascular morphology/function; Tumor spheroid response	Partial	Synergy combines several simultaneous vascular and tumor effects	Vascular protection combined with chemotherapy contributes under the tested conditions; important competing explanations remain.
Nicolas et al. (2022)	Exposure: Not applicable to vascular delivery; Target engagement: Pathway/state readouts reported	CAF state and senescence; ECM accumulation and treatment response	Yes	Principal stromal alternatives directly tested across interventions	Treatment-induced inflammatory stromal resistance is distinguished from the principal tested alternatives within the experimental context.
Hachey et al. (2023)	Exposure: Therapy delivered within vascularized microtumor; active local exposure not fully resolved; Target engagement: No	Vascularized response imaging/viability; Molecular or clinical-response comparison	Partial	Multiple co-varying patient-specific compartments remain	Patient-specific vascularized treatment response and TGF-β-related effects are supported under the tested conditions; important competing explanations remain.
Kim et al. (2023)	Exposure: Route-specific exposure studied; material loss and local active concentration not independently quantified; Target engagement: No	Tumor viability/proliferation; Secretome and gene-expression profiling	No	Transport restriction and endothelial signaling remain partly unresolved	Endothelial barrier versus endothelial-conditioned response contributes under the tested conditions; important competing explanations remain.

Continued on next page

Study	Effective/local exposure and target engagement	Orthogonal readouts	Rescue/reversal	Principal alternatives tested	Supported interpretation
Wisdom et al. (2023)	Exposure: Not applicable to drug exposure; Target engagement: Not applicable	T-cell adhesion and arrest; Extravasation and migration measured separately	Mechanism-blocking reversal; not restoration of treatment efficacy	The principal access step is directly tested; killing and persistence remain outside the model	Endothelial control of T-cell adhesion and migration is distinguished from the principal tested alternatives within the experimental context.
Farin et al. (2023)	Exposure: Direct culture exposure; vascular delivery not modeled; Target engagement: Molecular target/pathway analyses reported	Transcriptomic fidelity and stromal state; Drug-response screening	Partial	No endothelial or vascular-entry alternative can be tested in this model	CAF-state-dependent therapy response contributes under the tested conditions; important competing explanations remain.
Dekkers et al. (2023)	Exposure: Not applicable to vascular entry; Target engagement: Target-context and cell-state analyses reported	Live 3D behavioral imaging; Transcriptomics of engineered T-cell states	No	Vascular access is absent; post-contact behavioral alternatives are compared	Post-contact engineered T-cell behavior contributes under the tested conditions; important competing explanations remain.
Offeddu et al. (2024)	Exposure: Spatial delivery and pressure/compaction measured; systemic pharmacokinetics not modeled; Target engagement: No direct molecular target-engagement requirement	Pressure and tissue compaction; Drug-delivery distribution and response	Partial	Biomechanical and vascular contributions remain coupled; no definitive rescue of the whole endpoint	Biomechanical limitation of intratumoral delivery contributes under the tested conditions; important competing explanations remain.
Quintard et al. (2024)	Exposure: Perfusion and tissue function demonstrated; no tumor drug-exposure question; Target engagement: Not applicable	Perfusion and vascular function; Organoid growth, maturation, and function	No	No tumor or treatment-failure mechanism is tested	Platform or structural feasibility for reproducible vascularized-organoid platform performance; no therapy-resistance mechanism tested.
Du et al. (2025)	Exposure: Vascular perfusion/functional delivery assessed; exact active local exposure and target engagement require confirmation; Target engagement: Partial	Vascular architecture and angiogenesis; Tumor migration/metastasis and therapeutic response	Partial	Tumor-cell state and vascular effects remain partly coupled	Tumor-specific vascularization, metastasis, and anti-vascular targeting contributes under the tested conditions; important competing explanations remain.
Skubal et al. (2025)	Exposure: Particle perfusion and fluorescein wash-in/wash-out are proxies, not therapeutic active exposure; Target engagement: No	Vessel patency/perfusion; Fluorescein transport/retention and vascular response	No	Tracer transport, endothelial barrier, and therapeutic exposure are not equivalent	Tumor neovascularization, perfusion, and vascular-targeted therapy contributes under the tested conditions; important competing explanations remain.
Maity et al. (2025)	Exposure: Direct drug exposure; vascular entry not modeled; Target engagement: Pathway expression/secretome assessed	Tumor spheroid viability; Secreted-factor and molecular response	Partial	Pathway association is supported, but a complete pathway rescue and vascular alternative are not established	Pericyte-associated temozolomide resistance contributes under the tested conditions; important competing explanations remain.

Continued on next page

Supplementary Table S2B (continued)

Study	Effective/local exposure and target engagement	Orthogonal readouts	Rescue/reversal	Principal alternatives tested	Supported interpretation
Bains et al. (2025)	Exposure: Cell delivery through an endothelial channel; no drug-exposure issue; Target engagement: Antigen/context readouts reported	CAR-T trafficking and extravasation; Tumor killing and immune-state readouts	No	Access and post-contact effects remain partly coupled	Vascular and macrophage-mediated suppression of CAR-T cells contributes under the tested conditions; important competing explanations remain.
Liu et al. (2025)	Exposure: Controlled cellular perfusion through the vascular compartment; Target engagement: Cell-state, metabolic, and tumor-response targets assessed	Trafficking/extravasation and tumor contact; Killing, cytokines, single-cell or metabolic profiling	Yes	Access, activity, and candidate reversible dysfunction are compared across orthogonal assays and in vivo validation	CAR-T vascular access, activity, and pharmacologically reversible dysfunction is distinguished from the principal tested alternatives within the experimental context.
Ro et al. (2025)	Exposure: Drug exposure studied; local active exposure not independently matched; Target engagement: No	Longitudinal spheroid/endothelial imaging; Secretome and expression analyses	Partial	Transport, endothelial signaling, and scale effects remain incompletely separated	Endothelial interaction and treatment response contributes under the tested conditions; important competing explanations remain.
Bang et al. (2025)	Exposure: No direct local active exposure or target-engagement measurement identified; Target engagement: No	Angiogenic sprouting/vascular morphology; Tumor-cell viability/response	No	Drug-delivery and direct tumor effects remain partly coupled	Angiogenic sprouting and vascular-targeted treatment response contributes under the tested conditions; important competing explanations remain.
Shin et al. (2025)	Exposure: Perfusion/active local exposure not independently established; Target engagement: No	Angiogenesis and vascular morphology; Tumor growth/drug-response and validation across model contexts	No	Barrier, perfusion, and transport alternatives remain	Tumor-angiogenesis interaction and anti-vascular drug response contributes under the tested conditions; important competing explanations remain.
Blackledge et al. (2026)	Exposure: No therapeutic exposure question; Target engagement: No	Vascular geometry and perfusability; Secretome/ECM and monocyte recruitment	No	No tumor construct and no direct therapy-rescue experiment	CAF-state-dependent vascular and immune function contributes under the tested conditions; important competing explanations remain.
Ruan et al. (2026)	Exposure: Perfusable network reported; local active drug exposure not independently quantified; Target engagement: No	Vascular morphology/perfusion; Real-time ELISA/biomarker and organoid response	No	Delivery, vascular normalization, and direct tumor effects remain coupled	Vascularized cervical cancer organoid response and anti-VEGF treatment contributes under the tested conditions; important competing explanations remain.
Dai et al. (2026)	Exposure: Drug access in a perfusable vascularized tumoroid reported; precise target engagement requires confirmation; Target engagement: Partial	Vascular morphology/perfusion and delivery; Tumoroid response plus molecular/clinical comparison	Partial	Clinical concordance, delivery, and direct tumor effects remain partly coupled	Patient-specific vascularized colorectal tumoroid drug testing contributes under the tested conditions; important competing explanations remain.

Continued on next page

Supplementary Table S2B (continued)

Study	Effective/local exposure and target engagement	Orthogonal readouts	Rescue/reversal	Principal alternatives tested	Supported interpretation
Sun et al. (2025)	Exposure: Not applicable to vascular entry; Target engagement: Immune and tumor inflammatory-response pathways assessed	T-cell state, proliferation, and cytokine response; Tumor killing and inflammatory-cytokine sensitivity	Yes	Post-contact dysfunction and tumor sensitivity are directly tested; vascular access is intentionally outside the model	Reversible post-contact CAR-T dysfunction is distinguished from the principal tested alternatives within the experimental context.

Supplementary Table S3A. Evidence-component definitions and counts in the focused appraisal.

Evidence component	Operational definition	Full set (n=25)	Core (n=18)	Boundary (n=7)
Relevant comparator + mechanism-directed perturbation + functional readout	A comparator differs in the proposed bottleneck, the proposed mechanism is experimentally manipulated, and a mechanism-relevant functional endpoint is measured.	21	15	6
Principal competing explanation directly tested	The main alternative capable of producing the same endpoint is measured or experimentally manipulated.	4	2	2
Rescue or reversal included	Restoration or reversal of the proposed failed step is demonstrated with an appropriate control.	3	1	2
Reproducibility-oriented feature or independent benchmark	Explicit multi-donor/multi-batch testing, independent repeats, predefined QC/acceptance criteria, or an independent experimental or clinical benchmark is reported.	14	9	5
Cross-site, prospective, threshold-based, or comparable fit-for-purpose validation	Validation is performed for a stated context of use using one or more of these qualification-oriented features.	0	0	0

Supplementary Table S3B. Study assignments for the evidence components.

Evidence component	Assigned studies
Relevant comparator + mechanism-directed perturbation + functional readout	Nashimoto et al. (2020); Hu et al. (2022); Nicolas et al. (2022); Hachey et al. (2023); Kim et al. (2023); Wisdom et al. (2023); Farin et al. (2023); Dekkers et al. (2023); Offeddu et al. (2024); Du et al. (2025); Skubal et al. (2025); Maity et al. (2025); Bains et al. (2025); Liu et al. (2025); Ro et al. (2025); Bang et al. (2025); Shin et al. (2025); Ruan et al. (2026); Dai et al. (2026); Blackledge et al. (2026); Sun et al. (2025)
Principal competing explanation directly tested	Nicolas et al. (2022); Wisdom et al. (2023); Liu et al. (2025); Sun et al. (2025)
Rescue or reversal included	Nicolas et al. (2022); Liu et al. (2025); Sun et al. (2025)
Reproducibility-oriented feature or independent benchmark	Shirure et al. (2018); Hachey et al. (2021); Nicolas et al. (2022); Hachey et al. (2023); Farin et al. (2023); Dekkers et al. (2023); Offeddu et al. (2024); Quintard et al. (2024); Du et al. (2025); Liu et al. (2025); Bang et al. (2025); Shin et al. (2025); Dai et al. (2026); Sun et al. (2025)
Cross-site, prospective, threshold-based, or comparable fit-for-purpose validation	None

Interpretation note. These fields are qualitative evidence components rather than an ordinal grading scale. A study may satisfy one component while leaving another unresolved. The counts describe the focused appraisal and do not estimate prevalence in the field.

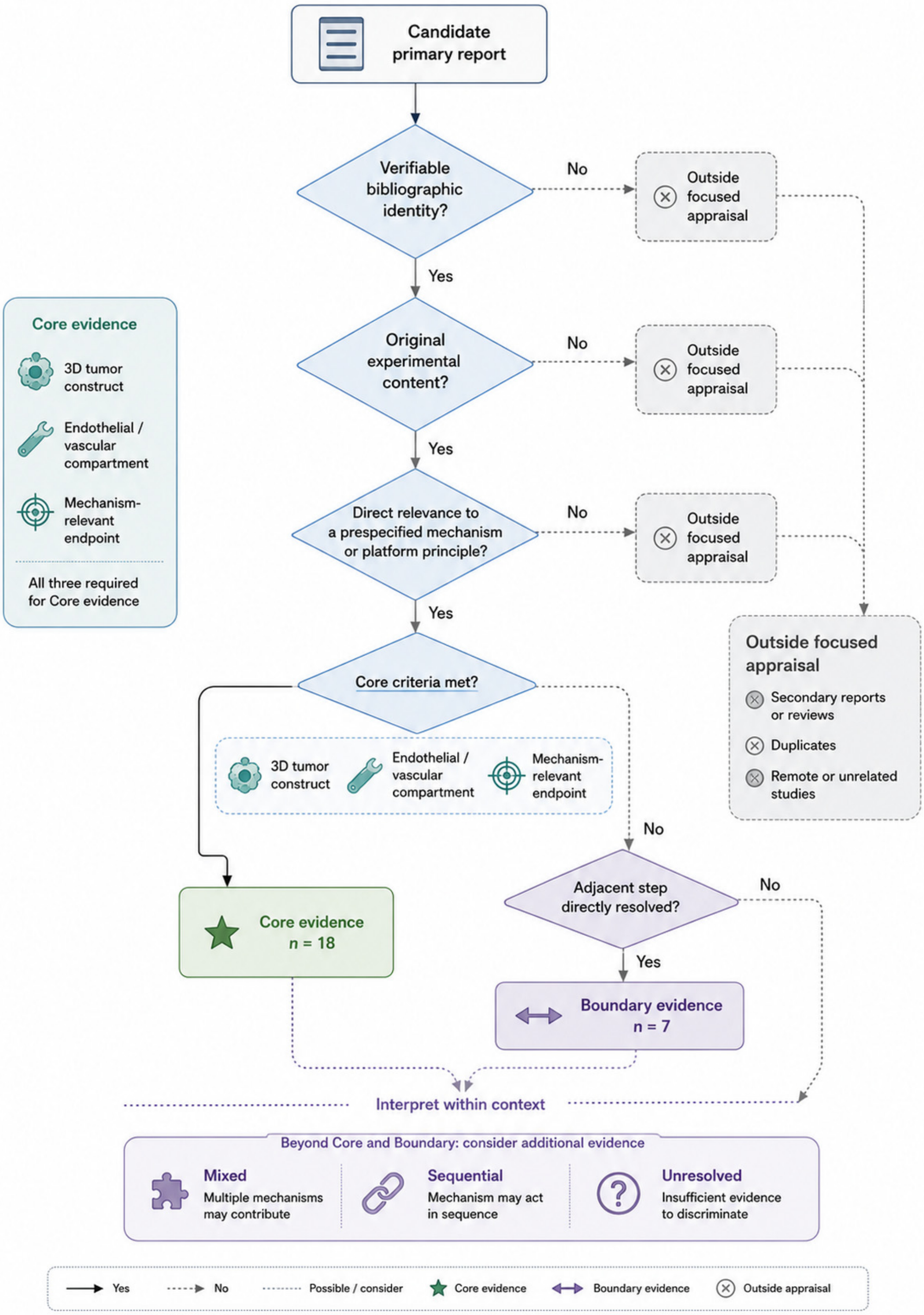

Figure S1. Scope-classification decision tree for the focused appraisal. Candidate primary reports required a verifiable bibliographic identity, original experimental content, and direct relevance to a prespecified mechanism or platform principle. Core evidence required all three model elements: a three-dimensional tumor construct, an endothelial or vascular compartment, and a mechanism-relevant endpoint. Reports that did not meet all Core criteria could enter Boundary evidence when they directly resolved an adjacent step; otherwise they remained outside the focused appraisal. Core and Boundary describe scope rather than study quality. The final focused set comprised 18 Core and seven Boundary studies. Mixed, sequential, and unresolved labels describe mechanism interpretation when available evidence does not support a single exclusive route.

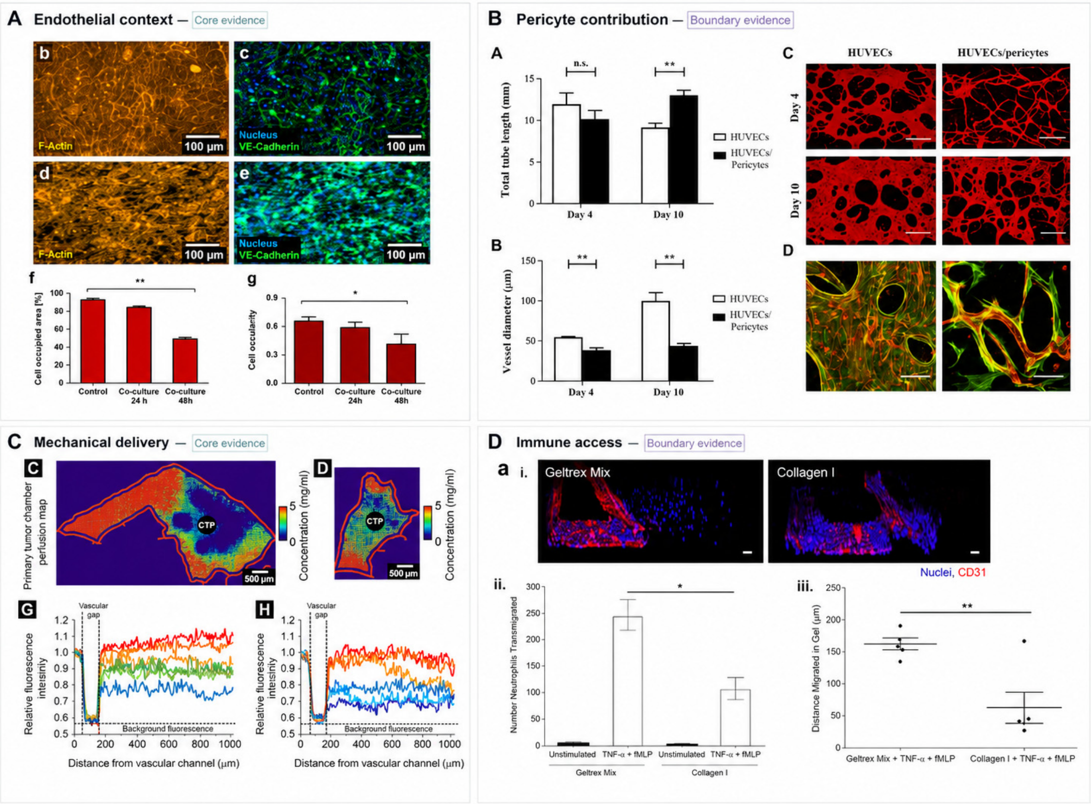

Figure S2. Representative published examples linking mechanism-relevant images to directly paired functional readouts. (A) Endothelial context: tumor-endothelium co-culture images and quantitative measures of endothelial integrity and circularity, adapted from Virumbrales-Muñoz et al. (2017), Figure 5b-g, DOI: https://doi.org/10.1038/s41598-017-12049-4, under CC BY 4.0. These panels support a context-dependent alteration of endothelial morphology and integrity but not universal endothelial resistance, local therapeutic concentration, or a distinct angiocrine mechanism. (B) Pericyte contribution: vessel length and diameter together with representative endothelial-pericyte co-culture images, adapted from Dibble et al. (2023), Figure 2A-D, DOI: https://doi.org/10.1038/s41598-023-31352-x, under CC BY 4.0. These panels support pericyte-associated changes in vascular morphology and perivascular localization but not tumor resistance or therapeutic delivery. (C) Mechanical delivery: tumor-chamber tracer-perfusion maps and spatial fluorescence-intensity profiles, adapted from Pradhan et al. (2018), Figure 4C,D,G,H, DOI: https://doi.org/10.1038/s41598-018-21075-9, under CC BY 4.0. These panels support architecture-dependent heterogeneity in tracer perfusion; tracer intensity does not establish therapeutic concentration, target engagement, or a unique resistance mechanism. (D) Immune access: three-dimensional reconstructions of neutrophil transmigration and paired measurements of transmigrated-cell number and migration distance, adapted from Riddle et al. (2022), Figure 3a.i-a.iii, DOI: https://doi.org/10.1038/s41598-022-10849-x, under CC BY 4.0. These panels support endothelial transmigration and matrix-dependent migration but not tumor-cell killing, post-contact immune function, or clinical efficacy. Panels were cropped, proportionally resized, arranged, and relabeled; original scale bars, color scales, axes, units, data points, error bars, and significance annotations were retained where present.

Supplementary material availability

Supplementary Tables S1A-S3B and Supplementary Figures S1-S2 are provided in this file. No new experimental or clinical dataset was generated.
